# Immunogenic cell death mediated TLR3/4-activated MSCs in U87 GBM cell line

**DOI:** 10.1016/j.heliyon.2024.e29858

**Published:** 2024-04-22

**Authors:** Seyed Mahdi Emami Meybodi, Fateme Moradi Moraddahande, Ali Dehghani Firoozabadi

**Affiliations:** a Yazd Cardiovascular Research Center, Non-Communicable Diseases Research Institute, Shahid Sadoughi University of Medical Sciences, Yazd, Iran; b Department of Medical Laboratory Sciences, School of Allied Medical Sciences, Shahid Beheshti University of Medical Sciences, Tehran, Iran

**Keywords:** Mesenchymal stem cells, Priming, TLR3, TLR4, Cell death, miRSeq

## Abstract

**Background and aims:**

Glioblastoma (GBM) is an aggressive primary brain cancer with no promising curative therapies. It has been indicated that MSCs can interact with the tumour microenvironment (TME) through the secretion of soluble mediators regulating intercellular signalling within the TME. TLRs are a multigene family of pattern recognition receptors with evolutionarily conserved regions and are widely expressed in immune and other body cells. MSCs by TLRs can recognize conserved molecular components (DAPMPs and PAPMPs) and activate signalling pathways, which regulate immune and inflammatory responses. MSCs may exert immunomodulatory functions through interaction with their expressed toll-like receptors (TLRs) and exert a protective effect against tumour antigens. As an emerging approach, we aimed to monitor the U87 cell line growth, migration and death markers following specific TLR3/4-primed-MSCs-CMs treatment.

**Methods and results:**

We investigated the phenotypic and functional outcomes of primed-CMs and glioma cell line co-culture following short-term, low-dose TLR3/4 priming. The gene expression profile of target genes, including apoptotic markers and related genes, was analyzed by qRT-PCR. MicroRNA-Seq examined the miRNA expression patterns, and flow cytometry evaluated the cell viability and cycle stages. The results showed significant changes in apoptosis and likely necroptosis-related markers following TLR3/4-primed-MSCs-CMs exposure in the glioma cell line. Notably, we observed a considerable induction of selective pro-apoptotic markers and both the early and late stages of apoptosis in treated U87 cell lines. Additionally, the migration rate of glioma cells significantly decreased following MSCs-CM treatment.

**Conclusion:**

Our findings confirmed that the exposure of TLR3/4-activated-MSCs-CMs with glioma tumour cells possibly changes the immunogenicity of the tumour microenvironment and induces immunogenic programmed cell death. Our results can support the idea that TLR3/4-primed-MSCs can lead to innate immune-mediated cell death and modify tumour cell biology in invasive and metastatic cancers.

## Highlights

• TLR3/4-primed-MSCs-CMs exhibit various properties at different doses and time points.
• TLR3/4-primed-MSCs-CMs decrease the viability of the U87 GBM-derived cell line.
• Low-dose treatment of TLR3/4 agonists increases glioma cell death and reduces cell migration.
• Necroptosis concept as a targeted therapeutic strategy for managing GBM.
• Sub-toxic inflammation or para-inflammation as a novel approach in programmed cell death.

## 1 Introduction

Gliomas are one of the most frequent primary intraparenchymal tumours, originating from the neuroglial stem or progenitor cells. The recently published categorization of the central nervous system (CNS) tumours has announced a new edition about classifying gliomas (neuronal and glioneuronal tumours). Glioblastoma (GBM-grade IV) is the most aggressive form of glioma that destroys normal brain tissue [1].

GBM risk factors are largely unknown, and diagnosis has been described as histopathological findings and molecular assessment, while only a little over 5 % of people survive for five years. The incidence rate of GBM is higher among men than women (60 % higher in males) and increases with age [2]. Comprehensive molecular profiling has lately been developed to achieve an accurate and personalized sub-classification of GBM. GBM is highly resistant to conventional therapy, including chemotherapy, radiation therapy, and aggressive surgery, due to microenvironmental crosstalk across multiple signalling pathways, tumour heterogeneity, and epigenetic/transcriptomic modifications of GBM [3]. Several mechanisms of action have been mentioned for glioma resistance, as well as DNA-repair enzymes, mismatch-repair (MMR) complex formation, glioma initiating cells (GICs), and tumour hypoxia that support tumour recurrences, progression, and therapeutic resistance [4].

In addition to the impermeable nature of BBB, conventional medication with chemotherapeutic agents is practically ineffective because of the limited penetration of anticancer drugs in solid tumours [5]. In this regard, different types of immunotherapy, including immune checkpoint blockade, oncolytic virotherapy, and vaccine therapy, have been offered to improve GBM management. Despite the progress in the field, GBM remains the most lethal tumour type, with limited therapy options and poor survival [6]. Considering the mentioned facts, new therapeutic approaches are needed to improve patient survival and effective clinical treatments to reinforce the tumour cell's sensitivity to chemotherapy.

Multiple cellular and molecular mechanisms of action, such as activation of the cell cycle components, disruption of programmed cell death, suppression of tumour suppressor genes and immune cells, activation of proto-oncogenes along with unidentified inflammatory conditions, and immune evasion by cancer cells are a long-running operation that contributes to the progression of tumour cells. The accumulation of genetic mutations activates proto-oncogenes, inactivates tumour-suppressor genes, and modifies epigenetic profiles, contributing to aberrant cell expansion and tumourigenesis [7].

We now understand that tumour evolution is driven by molecular alterations and genetic mutations that lead to inappropriate cellular proliferation. Ongoing research has provided a broad conceptual framework to manage cancer initiation and progression to examine how potential therapeutic opportunities might be optimally applied [8].

Many human cells perpetually devour themselves to defend the body against pathogenic substances and conserve dynamic equilibrium. In contrast, tumour cells evade death, which results in therapeutic resistance and tumour relapse. Programmed cell death (PCD) is ubiquitous in living organisms and is essential for tissue homeostasis by clearing aberrant cells. GBMs display a deregulated apoptotic pathway characterised by high levels of anti-apoptotic family proteins, leading to apoptosis inhibition and protecting normal and neoplastic cells from toxicity. Previous studies reported various missense and splice site mutations in particular genes, including p53 and DNA-binding domains, underlying escape mechanisms from apoptosis. Additionally, GBM resistance to apoptosis can be due to the over-expression of inhibitors of apoptosis (Bcl-2, BIRC1, and BCL2L2) and the suppression of apoptosis inducers (BOK, PMAIP1, caspase-6, caspase-7, caspase-8, APAF1) [9]. Thus, targeting cell death pathways by apoptosis is a potential target for therapeutic intervention [10]. Recently, the regenerative medicine arena, including tissue engineering, cell therapy, and gene therapy, has gained interest as a promising approach to cancer treatment. The use of stem cells and their related derivatives, such as cell-free compounds, in cancer management is one of the latest and most non-invasive approaches for cancer therapy [11]. Stem cells are recognized as their most impressive intrinsic features, such as self-renewal capacity and multi-lineage differentiation potency [12]. MSCs can be derived from different sources, including bone marrow, placenta, umbilical cord, peripheral blood, and adipose tissues [13,14].

MSCs have attractive properties that make them a suitable source for clinical practice, such as tropism to tumour sites and homing to tumour locations, anti-tumour characteristics, and suppression of tumour cell proliferation and metastasis. Some studies indicated that MSCs could mobilize and migrate toward tumour areas and suppress tumour growth by secreting several biomolecules, such as growth factors, chemokines, and cytokines in the TME and alter the nature of the TME that eventually promote apoptosis and inhibit the proliferation of tumour cells [[7], [8], [9]]. Even though the therapeutic effects of MSCs are controversial, some studies have indicated that they have an anti-tumour effect by inhibiting proliferation-related signaling pathways, such as the PI3K/AKT pathway, which inhibits cancer growth [15].

Moreover, studies revealed that MSCs display tumour-specific tropism and communicate with tumour cells via the effects of direct contact or paracrine factors. However, its underlying molecular mechanisms remain unclear and must be fully understood [16].

MSCs have some advantages over other stem cells: They are accessible, express a broad spectrum of multipotency and pluripotency markers, and over-express various types of cell adhesion molecules (CAMs), such as integrins in response to various cues. Additionally, they can be maintained and expanded in culture for long periods without losing their differentiation phenotypes.

In addition to the positive features of MSCs in cancer treatment, MSC secretion, commonly known as secretome, can activate the immune response and regulate TME. On the other hand, the secretory profile of MSCs secretome is tunable, engineerable, and can be modified by various approaches. Recent studies showed that MSCs secretome exerts an immunomodulatory effect by inhibiting/activating (immunomodulation) both the innate and adaptive immune system [17]. Over the past decade, it has been tried to discover the impact of MSCs-conditioned medium (MSCs-CM) on TME. Accordingly, the secretome of the MSCs contains a variety of paracrine factors gradient (peptide, protein, RNA, and lipid mediators) and MSC-derived extracellular vesicles (MSC-EVs) that influence TME and suppress proliferative and progression cues that eventually arrest tumour cell growth. However, previous studies have obtained inconsistent or even contradictory results on the therapeutic effects of MSC-CM [18,19].

MSCs may exert immunomodulatory functions through interaction with their expressed toll-like receptors (TLRs) and modulation of immune responses. TLRs are a class of pattern recognition receptors (PRRs) widely expressed by different immune cell populations to identify pathogen-associated molecular patterns (PAMPs). The evidence showed that TLRs are also naturally transcribed on different non-immune cells, such as MSCs, to protect them against infection [20]. The expression of TLRs, as well as TLR3 and TLR4, trigger an immune response through the recruitment of specific adaptor molecules, leading to NF-kB activation and pro-inflammatory cytokines secretion such as TNF-α and interferon-gamma (IFN-γ) [21].

Targeting TLRs indicates a promising field based on their power to activate innate and even modulate adaptive responses for a long-term protective effect against tumour antigens. Many compounds have been developed and used as a direct anti-tumour agent or combined with other common strategies to deal with different tumours [22]. These TLRs have been frequently used as a potent adjuvant for tumour vaccines or combined with conventional therapies and emerging immunotherapies [23,24]. Various preclinical and clinical trials administered intratumoural delivery of TLR agonists, which were shown to stimulate anti-tumour immune responses and tumour regression. A recent clinical trial revealed that the standard GBM therapy incorporated with the administration of HSPPC-96, as a TLR4 agonist derived from a patient's tumour, potentiated infiltration of CD8^+^ and CD4^+^ T cells in the injected tumours and upregulated the immune-related genes [25,26].

Preconditioning/priming approaches were recently proposed to modify MSC traits and enhance their therapeutic efficiency for different clinical applications [27]. Recent studies demonstrated enhanced immunomodulatory potential of MSCs after priming with pharmacological drugs and other physical and chemical agents. Several priming approaches have been designed to examine the possible effects of various physical or chemical agents on the characteristics and function changes of MSCs.

It has been revealed that different exogenous biomolecules can polarize MSCs into two subgroups, MSC-1 cells (pro-inflammatory phenotype) and MSC-2 cells (immunosuppressive phenotype), which can be induced by treatment with lipopolysaccharide (LPS) and polyinosinic-polycytidylic acid (Poly (I:C)), respectively.

There is a linkage between TLRs and the immunomodulatory phenotypes of MSCs, as previous studies showed that MSC preconditioning with TLR ligands, such as Poly (I:C) and LPS priming, leads to immunomodulatory modification of MSCs in various concentration and time exposure manner [28]. Previous studies reported that TLR4 activation on MSCs promotes their transformation into the MSC1 (pro-inflammatory) state. On the other hand, the TLR3 pathway enhances their immunosuppressive functions and polarizes them into the MSC2 anti-inflammatory phenotype [29]. The latest findings showed the therapeutic efficacy of TLR3/4-primed-MSCs in vitro and in vivo. For instance, TLR3-activated-MSCs shifted Th1 to Th2 and increased IL-10 production, resulting in colitis mice's improved survival rate. It is also described by the fact that the TLR3 priming promotes the homing of MSCs to the inflammatory sites in vivo and enhances their efficacy [30,31]. Moreover, a recently published study announced the anti‐tumourigenic effects of TLR4‐primed-MSC on pancreatic ductal adenocarcinoma cells via TNF‐α and IFN‐γ signaling pathways [32]. The anti-tumour property of MSCs after priming with LPS and Poly (I:C) is controversial and may depend on the routes of exposure, as we may observe a wide range of results at different routes of exposure.

As a substitute for naive MSC management, the preconditioning of MSCs is an auspicious therapeutic approach to potentiate the therapeutic effect of MSCs. Previous studies showed that TLRs-primed MSC condition medium could change the functional properties of MSCs, resulting in contrasting effects on the immune response and opposite roles in cancer progression (supportive or suppressive). TLR stimulation has been reported to cause cellular polarization, resulting in changes in the secretome, migration, and immunomodulation [33]. TLR3/4 priming of MSCs may reprogram them toward an anti-tumour phenotype and modulate the immune-mediated killing of tumour cells by up-regulating pro-inflammatory factors. It is linked to the activation of immune cells by MSCs in vitro and the retardation of tumour cells [34].

The primary goal of the present study is to assess controversial results regarding previous studies about the anti-tumour effect of MSCs and, more precisely, examine whether equipped MSCs with TLR3/4 agonists can potentiate the anti-tumour effect of MSCs. In our study, for the first time, we assessed the impact of TLR3/4-primed-MSCs-CMs on anti-tumour activities of MSCs in a time and dose-dependent manner by focusing on induced-apoptotic components cell signalling pathways and compared MSCs phenotypes (MSC-1 and MSC-2) on the GBM cell line. We revealed that MSCs-CM derived from TLR3/4-activated-MCSs impact the U87 cell line differently at various concentration-exposure time models. This paper demonstrated that an MSC-CM stimulated with TLR agonist significantly induces cell death in GBM tumour cells by regulating and modulating inflammatory-mediated programmed cell death. The results provide a theoretical base for new high-tech regimen therapy for GBM and lay the foundation of regenerative-based therapy development for brain tumours, which targets cross-signalling between inflammatory, apoptotic, and necroptotic signalling pathways.

## 2 Material and methods

### 2.1 Isolation, culture, and phenotyping of human-MSCs (h-MSCs)

The h-MSCs line S-1939 was sourced from Royan institute, Iran. They were cultivated under standard culture conditions in a complete stem cell medium. We cultured MSCs in low-glucose DMEM enriched with 10 % fetal bovine serum (FBS), penicillin/streptomycin (100U/100 μg/ml), and antifungal antibiotic amphotericin B agent at 37 °C, in a humidified atmosphere of 7,5 % CO2 [35]. The media was replaced every 2–3 days and used in passage 4. MSCs are characterized for CD44, CD73, CD90, and CD105 cell surface markers using high-throughput flow cytometry [36]. Adipogenic and osteogenic differentiation potency of MSCs was assessed by oil red and Alizarin red staining, respectively [37].

### 2.2 Priming TLR3 and TLR4 in h-MSCs

For TLR activation, MSCs were incubated with different concentrations of two small molecules with known bioactivity, poly (I:C), which is a TLR3 agonist, and LPS, consisting of a lipid and a polysaccharide of bacterial toxins (TLR4 agonist) in the dose and time-dependent manner (Table 1). We caused sterile inflammation by LPS small molecules and anti-inflammatory states following poly (I:C) preconditioning. Consequently, cells were rinsed with PBS, and the media was changed with DMEM low Glucose overnight [38].

**Table 1:** Dose and time exposure manner of MSCs preconditioning.

Agonist	Dose (concentration)	Time (duration of exposure)
LPS	10 ng/ml, 1 μg/mL, 10 μg/mL	.5 h, 1 h, 4 h
poly (I:C)	1 μg/mL, 2.5 μg/mL, 5 μg/mL, 10 μg/mL, 20 μg/mL, 50 μg/mL	1 h, 4 h, 6 h, 12 h, 18 h, 24 h

### 2.3 Preparation of h-MSCs conditioned medium (CM) and U87 cell line cultivation

The conditioned medium (CM) containing secreted factors from TLRs-primed-MSCs were collected after definite dose and time exposure. To collect the CMs, once the cell culture reached 90 % confluency (4th passage of MSCs), MSCs were preconditioned with different doses of TLR agonist. CM was centrifuged at 500×*g* for 5 min to eliminate cells and passed through a 0.22 μm syringe filter [39]. U87 cells were seeded into 24-well in DMEM medium containing 10 % of FBS and 1 % Pen/Strep (Gibco, Invitrogen Corporation, Grand Island, NY) and incubated at 37 °C and 5 % C. After that, media was aspirated and treated with different groups of TLRs-primed-MSCs-CM for 24 h, 48 h, and 72 h. Medium-containing DMEM and 10 % FBS were considered as the control group.

### 2.4 Gene expression analysis

After priming with a specific agonist, the mRNA expression levels of TLR3 and TLR4 were assessed by reverse transcription-quantitative polymerase chain reaction (RT-qPCR). After priming with different doses of LPS and poly (I:C), total RNA was isolated from the MSCs, cDNA was synthesized and followed by RT-qPCR [40]. Consequently, after the co-culture of the U87 cell line with different dilutions of TLRs-primed-MSCs-CM, the putative link between primed-CMs-induced gene expression profile changes was evaluated by RT-qPCR. In this regard, treated cells were trypsinized and collected for RNA extraction. TRIzol reagent (Invitrogen, #Cat no: 15596018) was used to extract total RNA based on the manufacturer's instructions. The quality assessment of extracted RNAs was performed on a 1 % agarose gel, and NanoDrop instruments were used to quantify the nucleic acid content in various samples (Thermo Scientific). Total RNA reverse transcribed using RevertAid First Strand cDNA Synthesis Kit according to the supplier guidelines (RevertAid First Strand cDNA Synthesis Kit, #K1622), and RT-qPCR reactions were carried out on Applied Biosystem (ABI) Step one plus using HOT FIREPol®. EvaGreen® qPCR Mix Plus (Solis biodyne, #Cat no: BIO-98005, Stonia) as described previously.

Specific primer pairs assessed the relative expression level, and GAPDH was considered the housekeeping gene (HKG) to normalize the reaction. NAC (No amplification control) and NTC (No template control) were involved in the reactions. The relative expression was computed based on the 2-ΔΔCT method. All reactions were done in duplicate. Primers used for qRT-PCR are itemized in Table 2.

**Table 2:** Primer sequences used for real-time quantitative PCR (qRT-PCR).

Genes	Primer sequences		Accession number	Product length (bp)
	Forward sequences (5′ to 3′)	Reverse sequences (5′ to 3′)		
TLR3	TCGAGAGTGCCGTCTATTTGC	TGGTGGTGGAGGATGCACA	NM_003265.3	175 bp
TLR4	ATATTGACAGGAAACCCCATCCA	AGAGAGATTGAGTAGGGGCATTT	NM_003266.4	300 bp
ActB	GCGTGACATTAAGGAGAAG	GAAGGAAGGCTGGAAGAG	NM_001101.4	172 bp
GAPDH	GAGCCACATCGCTCAGACAC	CATGTAGTTGAGGTCAATGAAGG	NM_001357943.2	150 bp
Bcl2	AGGCTGGGATGCCTTTGTGG	TGCATATTTGTTTGGGGCAGGC	NM_000633.3	173 bp
Caspase-3	TGAGGCGGTTGTAGAAGAGTTT	TTTATTAACGAAAACCAGAGCGCCG	NM_001354777.2	154 bp
Caspase-8	ATAGACTGGATTTGCTGATTACCT	CGGCAGAAGTGGAACCTGTA	NM_001228.5	112 bp
Caspase-9	GGATTTGGTGATGTCGGTGC	ACAGTCGATGTTGGAGCCAG	NM_001229.5	159 bp

### 2.5 miRNA-seq profiling

RNA extracted from the pool selective of TLRs-primed-MSCs-CM (Poly (I:C), 5 μg/mL; LPS, 10 ng/ml) applied for miRNA-seq. TruSeq Small RNA Library Prep Kit (Illumina, USA) was used to construct miRNA libraries compatible with Illumina multiplexing [41]. For bioinformatics analysis, we used the miRBase reference database for miRNA alignment (http://www.mirbase.org). Reads with low QC were excluded, and the qualified reads were aligned to the human reference sequence. The differentially expressed miRNAs were identified using bioinformatics analysis between untreated and TLR3/4-primed-MSCs-CM-treated U87 cell lines. The miRNAs with FDR P < .05 were considered significantly different.

### 2.6 Assessment of apoptosis and cell cycle distribution using flow cytometry

To confirm whether the TLR signalling pathways are associated with the induction of cell death, we performed annexin V/propidium iodide double-staining followed by flow cytometry analysis [42]. A panel of nine primed CMs experimental groups was used for the U87 cell line. In this regard, MSCs were seeded into 24-well plates and then co-cultured with selective doses of small molecules (CMs) for 4 h. The selective doses were as follows: Poly (I:C) (Sigma Aldrich P9582), 5 μg/mL; LPS (Sigma Aldrich, Cat number: L2880), 10 ng/ml (sub-toxic concentration); TAK-242, a specific inhibitor of Toll-like receptor 4 signalling (Sigma Aldrich, Cat number: 614316), 1 μM; Chloroquine disulphate (Sigma Aldrich, Cat number: C6628), 20 mg/mL; BAY 11–7082(Cell signaling. Cat number: 78679S), 10 nM; PX-478 (Active Bioscience), 5 μM after the treatment, The CMs harvested from the treated groups were added to U87 plates.

Consequently, the U87 cells were incubated for 48 h and rinsed twice with cold PBS. Subsequently, the specimens were suspended in an incubation buffer of 100 μl at one million cells/ml concentration. To determine the presence of phosphatidylserine on the cellular membrane, a volume of 2 μl of Annexin-V-Flous was added to tubes. The tubes were then incubated in darkness for 20 min. The fluorescence emitted from the sample was quantified using flow cytometry, providing evidence of the interaction between Annexin-V-Flous and the phospholipid. During the experiment, PI staining was used to examine cell distribution at various cell cycle stages. Different groups were given a treatment consisting of 70 % ethanol throughout a length of time that was specified to last for 24 h. In order to get precise staining of the DNA, the cells that had been fixed were first put through treatment with RNase and then exposed to PI. This was done so that the staining would be correct. Flow cytometry and the software Windows FlowJo V10 were used to quantify the distribution of cells throughout a number of stages of the cell cycle, consisting of G1, S, G2/M, and sub-G1.

### 2.7 Scratch-wound assay

The scratch assay evaluated the wound closure activity of TLRs-primed-MSCs-CMs on U87 cell lines [43]. Scratched wound lines were created upside the U87 cell line using a sterile yellow micro-tip (200 μl), and PBS discarded the detached cell. Then, the cells were supplemented with various dilutions of TLRs-primed-MSCs-CM. Cell motility and wound healing area ( × 100) were detected and photographed with an inverted light microscope (Olympus, BX51, Japan) at definite incubation times. Wound healing was measured using the PixelProTm 3.0 LABOMED, USA different sites from each scratched area of gaps. The migration area ratio = (Ao – An)/Ao; Ao: represented the initial wound area (t = 0 h); An: represents the residual area of the wound at the assess point (t = D h).

### 2.8 Statistical analysis

The statistical software GraphPad Prism 9 (InStata, GraphPad Software Inc) was applied to accomplish statistical operations [44]. Results are given as mean ± SEM (standard error of the mean) or mean ± SD, as indicated. Statistical significance was evaluated with the Student's t-test (between two groups) and one-way ANOVA and nonparametric method (Kruskal-Wallis test) (for more than two groups) using the uncorrected Fisher's LSD test. The level of significance was denoted with an asterisk corresponding to the p-value (*: p < 0.05, **: p < 0.01, ***: p < 0.001, ****: p < 0.0001).

## 3 Results

### 3.1 Characterization of MSCs

**Fig. 1:**
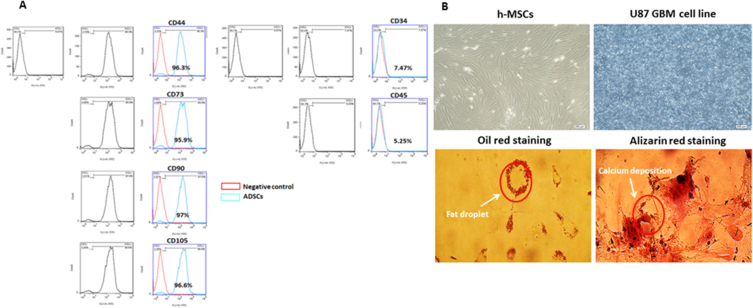
A) Schematic representation of four classical cell surface markers expressed in MSCs by enzymatic digestion method was validated by flowcytometry among freshly isolated and expanded MSCs. Illustrative histograms of surface marker expression compared to unstained MSCs (negative control). MSCs expressed CD44, CD73, CD90 and CD105, but did not express CD34 and CD45. B) MSCs have a spindle and fibroblast-like shape, illustrating the typical shape of MSCs and the U87 cell line has an epithelial morphology. Cells differentiated for 12 days have altered morphology and contains lipid drops. Differentiated cells stained with Oil Red O had well-defined lipid inclusions. Phase contrast microscopy allowed us to observe large lipid drops. Oil red O staining shows cells grown in the defined inductive medium differentiate into adipogenic lineages, for osteogenic differentiation induction was validated by alizarin red staining. Cells grown in a defined inductive medium differentiate into osteogenic lineages and finally various calcium depositions were seen. (For interpretation of the references to color in this figure legend, the reader is referred to the Web version of this article.)

Expression and confirmation of four classical cell surface markers expressed in MSCs were validated by flow cytometry among freshly isolated and expanded MSCs. MSCs expressed the primary cell surface markers of MSCs CD44, CD73, CD90, and CD105, but we reported a lack or shortage of the expression of human hematopoietic markers, CD34 and CD45 (Fig. 1-A). The percentage of positive cells for classical markers was assessed after the withdrawal of the non-specific fluorescence signal with the isotype-matched control antibodies.

### 3.2 Multilineage differentiation potential of MSCs

#### 3.2.1 Differentiation into adipogenic lineage

The MSCs (n = 5) were first grown to>90 % confluence and incubated in the induction and maintenance media. They were then fixed and stained with Oil Red O to detect fat deposition. The MSCs were differentiated into adipocytes when grown in adipogenic induction media. Once the adipogenic induction media was added, cell proliferation stopped, and the cells appeared flattened and increased in size (Fig. 1-B)

#### 3.2.2 Differentiation into osteogenic lineage

Osteogenesis was validated using von Kossa and alizarin red S staining to show ECM calcification; we detected alizarin red S-calcium complex formation, observing a bright red stain.

### 3.3 Real-time PCR gene expression profiling

**Fig. 2:**
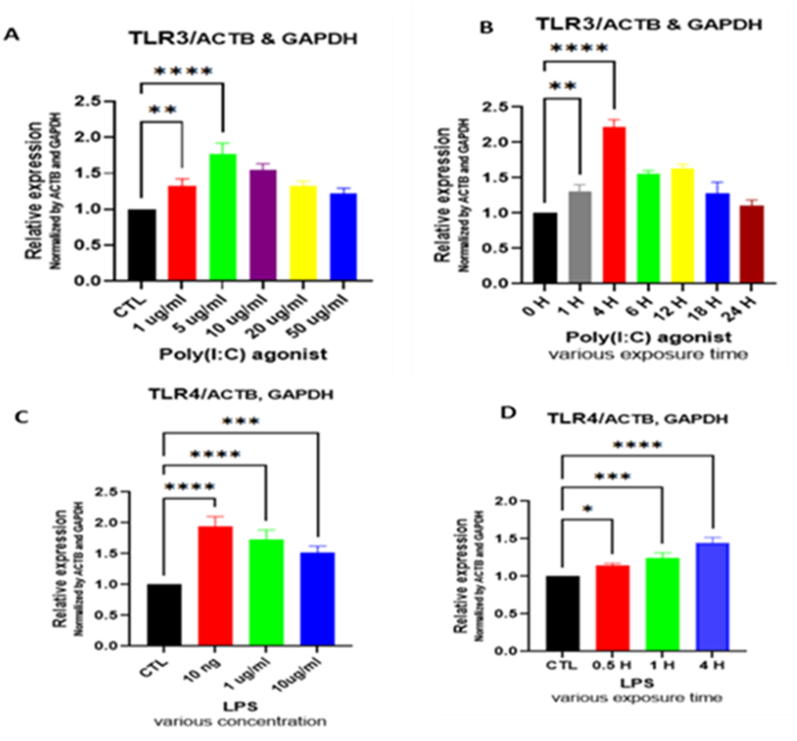
The expression level of TLR3/4 genes in MSCs elevated following exposure to LPS and Poly (I:C) at various dosages/times exposure. A) Expression level of TLR3 following Poly (I:C) exposure in a dose-dependent manner. B) Expression level of TLR3 at different exposure time points. C) Expression level of TLR4 following LPS exposure in a dose-dependent manner. D) Expression level of TLR4 at various time points following exposure to LPS. Values depicted are mean ± SD from 3 independent cell cultures and duplicate repeated RT-PCR. The GAPDH and β-actin were used to normalize data in qRT-PCR. ANOVA with Fisher's least significant difference (LSD) was applied to compare gene expression levels (*P < .05; **P < .01; ***P < .001; ****P < .0001).

Firstly, we determined the expression level of TLRs in primed MSCs. In this regard, we used specific primers to detect the expression levels of human TLRs from total RNA. The results showed that primed MSCs significantly overexpressed TLR3 and TLR4. Based on the results, the highest expression levels of TLR3 and TLR4 were observed in 5 μg/mL and 10 ng/ml concentration at 4 h exposure time, respectively (Fig. 2).

**Fig. 3:**
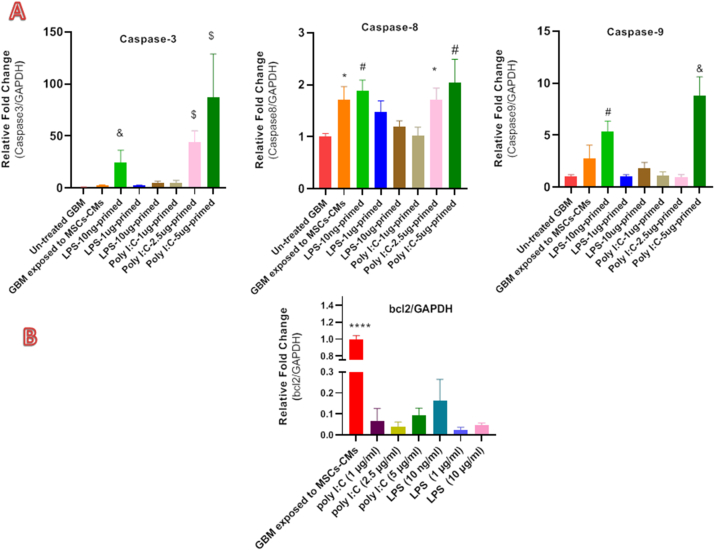
A) Generally, the results showed the increased caspase-3 mRNA levels in GBM exposed to TLRs-primed groups. U87 cells pretreated with 1, 2.5, and 5 μg/ml poly (I:C) primed MSCs-CM significantly increased caspase-3 expression level (2.5 and 5 μg/ml ****P < .0001). Also, we observe significantly enhanced expression in the TLR4 primed group at LPs 10 ng/ml (***P < 0. 001). Meaningfully, a light correlation between TLR3, TLR4, and caspase-3 mRNA expression was observed in the U87 cell line. These observations demonstrate that TLR3 and TLR4 activation possibly drive cell death in tumour cells. Our results confirmed in vitro overexpression of caspase-3, a key mediator of apoptosis. Similarly, the expression of caspase-8 and caspase-9 at the mRNA level, strongly related to the apoptosis signalling pathway, was elevated at specific concentrations of TLR agonists (*: p < 0.05, #: p < 0.01, &: p < 0.001, $: p < 0.0001). B) The Bcl-2 expression significantly decreased in selective groups. Apoptosis is regulated by key regulators of anti-apoptotic and pro-apoptotic family members of the B cell lymphoma 2 (Bcl-2). Bcl-2 can inhibit apoptosis by preserving the outer membrane integrity. All the primed groups considerably decreased the expression level of Bcl-2 in U87 cell line (****P value < .0001).

The RT-qPCR measured the in vitro anti-proliferative and apoptosis-inducing effect of TLRs-primed conditioned medium on the U87 cell line. The analysis of large sets of genes revealed differential expression in the mRNA level of markers associated with apoptosis in treated glioma cell lines compared to the untreated group. The expression levels of selective pro-apoptotic genes such as caspases 8, 9 and 3 were significantly increased in treated groups. On the other hand, the expression level of the anti-apoptotic gene Bcl-2 was much lower in the treated U87 cell line. Our results showed that various concentrations of TLR agonists significantly decreased the Bcl-2 expression level (p-value: <0.0001) in the U87 cell line (Fig. 3).

### 3.4 Differentially expressed miRNAs

**Fig. 4:**
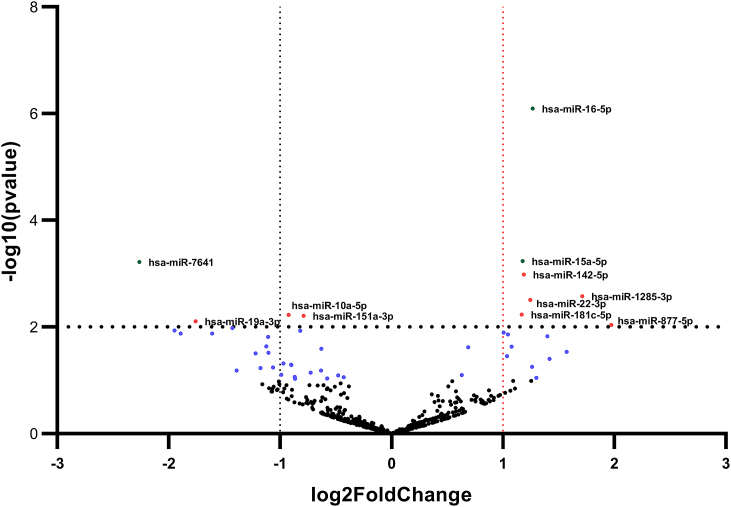
Volcano plot presenting varied miRNA expressions detected using miRNA-seq in the U87 cell line. The y-axis shows the log 10 of the *P*-values, and the x-axis is the FC (calculated as the log-ratio transformed ratio of the expression between TLR3/4-primed and unprimed groups). miRNAs with logFC < − 1 and logFC >1 (with a p-value <.05) are displayed in green. The green and red labels show the top miRNAs with high levels of dysregulation. Unique DE miRNAs (hsa-miR-16-5p, hsa-miR-15a-5p, and hsa-miR-7641), whose targets were found to be associated with cell survival and apoptosis have been identified in treated U87 cell line. (For interpretation of the references to color in this figure legend, the reader is referred to the Web version of this article.)

The volcano plot illustrating the differentially expressed miRNAs is shown in the figure below (Fig. 4). It correlates the magnitude of changes (FC, x-axis) and statistical significance (*P*-value, Y-axis). Interestingly, we identified that hsa-miR-15a-5p, hsa-miR-16-5p, and hsa-miR-7641 were significantly dysregulated in the U87 cell line.

In addition to the mentioned miRNA, miR-142-5p, miR-1285-3p, MiR-181c-5p, and MiR-877-5p were significantly up-regulated in the U87 cell line after treatment. Many studies stated that they function as a tumour suppressor and mitigate tumour progression through different signalling pathways.

### 3.5 Flow-cytometry

**Fig. 5:**
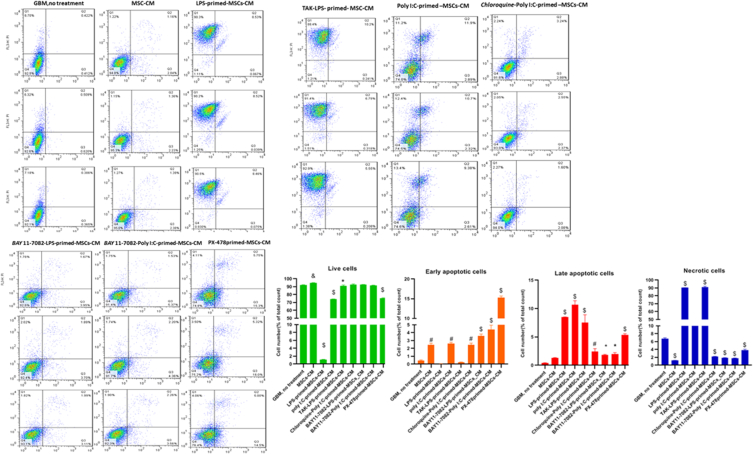
Combination of imaging flow cytometry and Annexin V/PI staining protocol used to detect cell death induced by TLRs-primed-MSCs-CMs for 48 h in the U87 cell line. The level of significance was denoted with an asterisk corresponding to the p-value (*: p < 0.05, #: p < 0.01, &: p < 0.001, $: p < 0.0001).

Cells were categorized into four distinct groups in flow cytometry analysis results: the lower right quadrant signifies necrotic cells (Annexin V-/PI+), the upper right quadrant indicates late apoptotic cells (Annexin V+/PI+), the upper left quadrant represents early apoptotic cells (Annexin V+/PI-), and the lower left quadrant of each panel show viable cells (Annexin V-/PI-). Cells stained with Annexin-FITC were considered apoptotic cells (upper region) (Fig. 5). We observed a considerable induction of both the early and late phases of apoptosis following TLRs-primed-MSCs-CMs treatment. Following LPS-primed-MSCs-CM treatment, the percentage of early apoptotic cells decreased significantly, while the number of late apoptotic and necrotic cells considerably increased in the U87 cell line. We applied TAK-LPS-primed- MSC-CM treatment to assess the effect of TLR4 antagonists on U87 cell lines. Surprisingly, TAK enhanced the number of live cells as giant as the control group and reduced early apoptosis in the cell line, even though it couldn't inhibit the necrosis phase of U87 cells. To assess the impact of NFKB in TLR4 involvement of apoptosis, we primed CM with BAY 11–7082 before LPS priming. Interestingly, we faced results that were precisely the opposite of LPS priming. So, we observed an increase in early apoptosis and a reduction in late apoptosis and necrotic cells.

Following Poly I:C-primed–MSCs-CM treatment, our results showed a significant rise in the number of early, late, and necrotic cells in the U87 cell line. It strongly confirms the role of the TLR3 signalling pathway in apoptosis. Meanwhile, pr-etreatment of Chloroquine, an inhibitor of endocytic TLR3, neutralized the effect of Poly (I:C) preconditioning and significantly reduced the number of late apoptotic and necrotic cells. Similarly, BAY 11–7082/Poly (I:C) co-treatment plunged the number of late apoptotic/necrotic cells.

Hypoxia-inducible factor-1α (HIF-1α) is severely reported as a critical factor for tumour progression. Previous studies demonstrated that tumour development and maintenance are supported by NF-κB, while cellular proliferation and adaptableness to angiogenic signals are assisted by HIF-1α [45]. In this regard, PX-478, an inhibitor of HIF-1α, was used to confirm the effect of HIF-1α blockage on U87 cell lines and its contribution in tumour progression. Notably, we witnessed an increase in both apoptosis phases and necrosis in the U87 cell line.

**Fig. 6:**
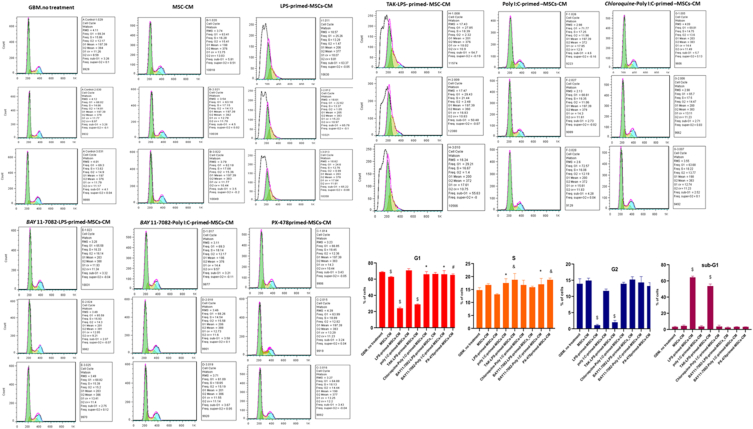
Cell cycle analysis and quantitative measurement of cell cycle phase through PI staining and following flow cytometry for the U87 cells following different CMs treatment (*: p < 0.05, #: p < 0.01, &: p < 0.001, $: p < 0.0001).

Cell growth inhibition was tested by measuring the distribution of the cell cycle using flow cytometry (Fig. 6). After the U87 cells were treated with different primed CMs, we analyzed the effect of various primed CMs on cell cycle distribution. We observed that after 48 h of incubation, LPS-primed-MSCs-CM strongly arrested cell cycle progression at the G2/M phase in U87 cells and significantly increased the sizes of the sub-G1 populations compared to the untreated group. At the same time, pre-treatment of TAK-242 increased the G2 phase and decreased the sub-G1 phase in the TAk 242-LPS-primed-MSCs-CM group. Also, pre-treatment of BAY 11–7082 increased the number of G2 phases and significantly dropped the sub-G1 population. On the other hand, we did not observe a strong association between Poly (I:C) preconditioning and cell cycle distribution even following pre-treatment of Chloroquine and BAY 11–7082. Interestingly, PX-478 primed-MSCs-CM increased the cell population at the G1/S phase but did not show significant modification in the G2/M and sub-G1 phase.

### 3.6 Scratch-wound assay results

**Fig. 7:**
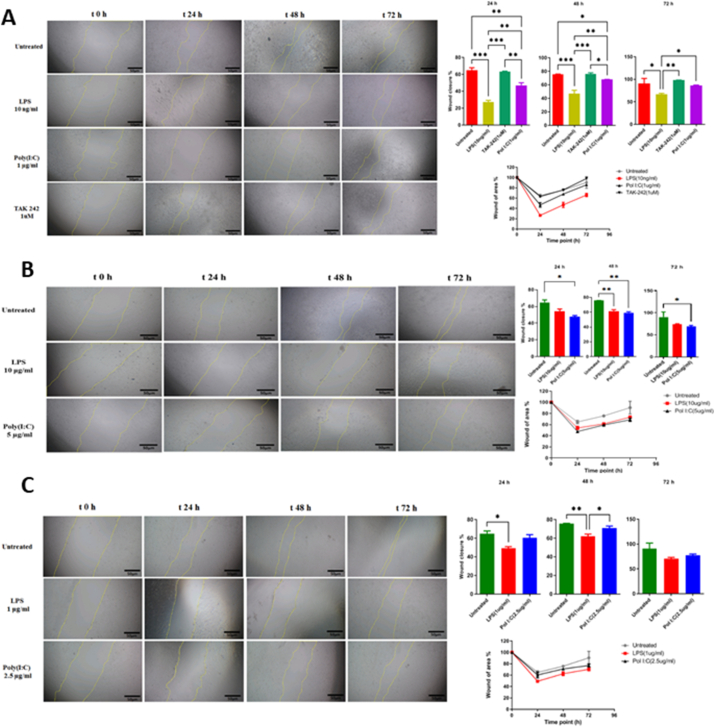
Wound healing assay to evaluate cell migration. The assay was performed at 0, 24, 48, and 72 h in TLRs-primed-MSCs-CMs treated and unprimed U87 cell lines. (A) Illustrative phase-contrast microscope images presenting the wound closure by the cells at 0, 24, 48, and 72 h and graph of scratch wound healing assay in untreated, LPS (10 ng/ml), TAK-242(1 μM) and Poly (I:C) (1 μg/ml) groups. (B) Representative images of scratch wound healing assay of Untreated, LPS (10μg/ml), and Poly (I:C) (5 μg/ml) groups. (C) Scratch-wound closure was monitored over time in Untreated, LPS (1 μg/ml), and Poly (I:C) (2.5 μg/ml) groups. Cell migration was measured by the rate of cells moving towards the scratched area upon time using ImageJ ™ software. Data were analyzed over time (vs. time = 0 h) using a one-way ANOVA followed by Uncorrected Fisher's LSD test (*P < .05 and * *P < .01, ** *P < .001).

Treatment effects on glioma cell line migration were determined using the wound-healing assay. The wound closure rate was compared between the U87 cell line groups with and without TLRs-priming. There was a significant reduction in cellular migration of U87 cancer cells at TLR3 and TLR4 preconditioning (Fig. 7). TLR-primed-MSCs-CM suppressed the motility of glioma cells. Meaningfully, we observed constrained glioma cell migration in LPS (10 ng/ml) and Poly (I:C) (1 μg/ml) doses. To confirm the TLR4 impact on glioma cell migration, we preconditioned MSCs with TAK-242(1 μM), a specific TLR4 signalling pathway inhibitor. Interestingly, it significantly enhanced the migratory potential of the mentioned group. In addition to the selective group, we used various doses of LPs and Poly (I:C) to precondition MSCs. Notwithstanding, our results generally showed that preconditioning could attenuate migration in different groups, and we achieved the most significant anti-migratory effect in recommended doses of LPS(10 ng/ml) and Poly (I:C) (1 μg/ml).

## 4 Discussion

GBM is mainly known as a sporadic, aggressive, and challenging type of adult brain cancer. Classical modalities, the most widely employed treatments to fight against cancer (surgery, chemotherapy, and radiotherapy), are the gold therapy for GBM. However, studies indicated that the activity of the antineoplastic drugs is greatly attenuated due to the restrictions imposed by the particular junctions of the BBB [46]. The insufficient and off-target effect of conventional therapeutic approaches has been a challenging problem in GBM therapy.

During the past decades, cell-based and immunotherapy have revolutionised cancer therapy. Chimeric antigen receptors (CARs), natural killers (NK) and stem cells have been the most frequently applied cell-based strategies to treat different types of cancer, ranging from solid tumours to hematologic malignancies [47]. Recently, research on the unique characteristics of stem cells has gained increasing attention as a potential treatment for the fight against cancer. Stem cells can release various paracrine factors and soluble materials, such as EVs, cell-derived membranous vesicles comprising multiple bioactive molecules, which may significantly impact tumour cells. MSCs belong to the adult stem cells (ASCs) group and are a promising treatment against cancer due to their specific feature. It was originally thought that they play their role directly, but it is now revealed that they exert therapeutic effects primarily based on their paracrine function. Multiple factors, including growth factors and cytokines, are secreted from MSCs, which can mediate various signalling pathways such as cell growth, angiogenesis, cell survival, cell death, and immunomodulation of cancer cells and significantly impact tumour progression. The released factors, collectively named secretomes, are directly released into the extracellular matrix or bundled into EVs to spread in the tumour microenvironment.

Over the last decades, stem cell manipulation strategies such as genetic engineering and priming have been fascinating approaches to supplying tumour-suppressor mediators into tumour cells. Previous studies served different viral and non-viral methods to modify the expression route of MSCs. Extensive studies reported genetically engineered MSCs with tumour necrosis factor-related apoptosis-inducing ligand TRAIL, BMP4, and PTEN efficiently suppressed glioma tumour growth and induced apoptosis [[48], [49], [50]]. Preconditioning MSCs with various physical and chemical cues has been a new approach to improving MSC's functionality. Some Studies indicated different behaviour of MSCs after priming with hypoxia conditions and clinical drugs [51].

Unregulated programmed cell death and the loss or mutation of tumour suppressor genes may lead to uncontrolled proliferation and tumour formation. Glioblastoma cells conserve proliferative potency by dysregulating pathways involved in senescence-like cell cycle arrest and PCD.

The apoptosis cascades have been divided into two central routes: the extrinsic or DR pathway, depending on the binding of the proper exogenous ligand, and the intrinsic mitochondria pathway, according to the origin of the death stimuli [52]. The processes of apoptosis are directed by a cluster of proteases entitled caspases, which have been classified into two main groups: initiators (caspase 2, 8, 9, and 10) and effectors (caspase 3, 6, and 7), according to their activation phase. Apoptosis pathways lead to the enzymatic activation of caspases and induction of biochemical and morphological modifications, including cell shrinkage, chromatin condensation, nuclear fragmentation, and membrane blebbing. Caspase-8 is an apical regulator of apoptosis. The initiation of cell death is through the activation of Caspase-8.

Consequently, it cleaves and drives downstream signalling, leading to cell death. Following caspase-8 activation, subsequent signalling stimulates several downstream effector caspases [3,6,and7]] and the execution phase of apoptosis by causing nuclear and cell shrinkage. On the other hand, caspase-9 plays a critical role in the intrinsic pathway of apoptosis. It is triggered by proteolytic cleavage at Asp-315 and Asp-330 sites, which stimulate the release of the active catalytic domain and the formation of an apoptosome, an essential scaffold in initiating apoptosis. It comprises caspase-3, caspase-9, and apoptosis-inducing factors such as cytochrome *c* (Cytc) [53]. Mitochondrial DNA (mtDNA) mutations can be diagnosed in approximately 60 % of solid tumours, and the accumulation of mtDNA mutations causes mitochondrial dysfunction. In GBM, mitochondrial function is impaired due to mtDNA mutations, resulting in variable-degree changes in morphology and abnormal bioenergetics, including overproduction of ROS. It leads to aberrant intrinsic and extrinsic pathways of apoptosis linked to tumour survival and cell death in GBM [54].

GBM indicates deregulation in programmed cell death, which enables GBM cells to gain multi-drug resistance (MDR). Thus, research into the possible modulation of GBM cell life or death is an enormous step toward achieving novel targets for therapeutic intervention [55].

GBMs display a deregulated apoptotic pathway characterised by high levels of the BCL-2 family proteins, leading to apoptosis inhibition and protecting normal and neoplastic cells from toxicity. Previous studies demonstrated the correlation between patients with Bcl-2 overexpression and shorter survival time. On the other hand, Bcl-2 overexpression is correlated with clinical and paraclinical parameters and tumour resistance against cytotoxic treatment [56]. Some studies revealed that gliomas might express high levels of Bcl-2 protein, possibly enabling GBM cells to resist and evade apoptosis [57,58]. Previous studies reported that apoptosis resistance in GBM cells could be due to the over-expression of inhibitors of apoptosis (Bcl-2, BIRC1, and BCL2L2) and the suppression of apoptosis inducers (BOK, PMAIP1, caspase-6, caspase-7, caspase-8, APAF1) [52].

**Fig. 8:**
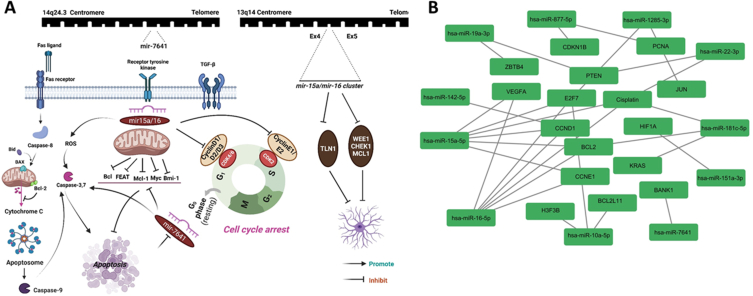
A) Interaction of mir15a/16-5p cluster and mir-7641 with targeted genes involved in intrinsic and extrinsic signalling pathways of apoptosis. As depicted above, the mir-15a/16 cluster contributes to apoptosis induction by suppressing anti-apoptotic genes such as Bcl-2 and Mc-1. Moreover, miR-16-5p probably inhibits glioma cell viability and invasion through miR-16-5p/Talin-1 (TLN1) Axis. It is worth mentioning that miR-15a and miR-16 can induce apoptosis and cell cycle arrest by suppressing cyclin D1 (CCND1). On the other hand, miR-7641, as an oncogenic miR, promotes tumour cell proliferation by improving intercellular communication. B) Network construction of top differentially expressed miRNAs (DE-miRNAs) and their target mRNAs.

Dysregulation of microRNAs is commonly associated with human malignancies and holds great promise as tissue-specific biomarkers of neoplasia. MiR-16-5p contributes to the enlargement of different malignancies, including neuroblastoma and brain tumours. This miRNA has 22 nucleotides and is transcribed by the miR-16-1 gene [59]. miR-16-5p, as a candidate tumour suppressor miRNA, is commonly suppressed in astrocytic gliomas and controls biological behaviours of glioma cells, including cell viability, apoptosis rate, and epigenetic modification of target cells [60]. Moreover, it has been shown that Tanshinone IIA, a traditional Chinese herbal, decreases survival and invasiveness and also reduce expression levels of matrix metallopeptidase 9 (MMP-9) and cyclin D1 through miR-16-5p/Talin-1 (TLN1) Axis [61]. Surprisingly, our results confirm the correlation between TLR activation and expression of miR-16-5p gene [62]. MiR-15a-5p, as a suppressor of tumour formation, promotes apoptosis and inhibits tumour growth by targeting specific oncogenes, such as Bcl-2, CcnD1, Mcl1, and Wnt3A [63]. It is reported that hsa-miR-15a-5p overexpression is a potent inhibitor against tumour cell proliferation and migration. It is supposed that hsa-miR-15a-5p is involved in crucial biological processes through its targeted gene, G1/S-specific cyclin-D1 (CCND1). Bioinformatics and dual-luciferase reporter assay analyses predicted CCND1 as a target of hsa-miR-15a-5p [64]. Additionally, a recent study revealed that miR-15a-5p promotes apoptosis and inhibits cell growth through the changes in expression levels of pro-survival genes and proteins, including Bcl-xl and Bcl-2 and inhibition of oncogenic transcription factors such as CCND2, Bcl-6, MYC, and PIK3R1 [65]. It is reported that miR-15a-5p induces lung cell apoptosis by activating caspase-3/9 in a monocrotaline (MCT) rat model [66] (Fig. 8).

Patel et al. illustrated that overexpression of miR-16 and miR-15a inhibits BMI1 levels and enhances ROS production in breast cancer cells, which causes a significant impairment in the mitochondrial membrane potential. They confirmed that miR-16 and miR-15a bind to the 3′ UTR of BMI1 transcript and regulate the expression level of BMI1. Moreover, they reported significant up-regulation of pro-apoptotic genes such as BID, BAX, caspase-9, caspase-3, and cytochrome-c and suppression of BMI1 and anti-apoptotic proteins including Bcl-2. Following miRNA transfection, anti-apoptotic protein Bcl-2 significantly down-regulated, whereas caspase-6/9 and caspase-3 were stimulated by the release of cytochrome C into the cytoplasm, resulting in apoptosis of BC cells [67]. Additionally, wound healing assay results revealed that cells having up-regulation of miR-16, miR-15a, and both miR-15a/16 indicated decreased migratory effect compared to scramble transfected cells. Our migration assay result was consistent with the Patel et al. study, and the decreased migration potency of U87 cells can be attributed to up-regulation of miR-15a/16.

A similar study has also reported that the expression of miR-15a/16 exerts its apoptotic regulation by targeting Bcl-2. Using bioinformatics tools displays miR-15/16, and the Bcl-2 mRNA includes a complementary site at the first nine nucleotides from the five ends of both miRNAs and 3′-end of the Bcl-2 transcript. In this regard, Bcl-2 can considered one of the top targets of posttranscriptional regulation by miR-15/16 cluster [68].

Taken together, prior investigations confirmed that dysregulation of miR-15a/16 cluster is closely related to the development of various cancers through taking part in different biological pathways such as cell proliferation, programmed cell death, angiogenesis, invasion, and metastasis. MiR-15a/16 cluster can suppress tumour cells in their microenvironment and increase the efficacy of chemotherapeutic drugs [69].

Notwithstanding the apparent anti-tumour role of miR-15a-5p, some studies reported the paradoxical role of miR-15a-5p in colorectal adenocarcinoma and T98G glioblastoma cells [70,71]. These controversies highlight the necessity of clarifying the putative role of miR-15a-5p in various tumours.

On the other hand, miR-7641 as another oncomir was found to be down-regulated in the treated U87 cell line. Several studies confirmed the tumour-promoting potency of the miR-7641 as one of the critical components of extracellular vesicles and exosomes. It stimulates various tumour growth and progression via intercellular communication. It can cause epigenetic changes in recipient cells following transportation by exosomes carrying miR-7641. Our result is consistent with previous literature on breast and gastric cancers [72,73]. In addition, Abu Musa Md. et al. showed that suppression of miR-7641 promotes doxorubicin-mediated apoptosis by enhancement of pro-apoptotic markers such as caspase-9 and poly ADP ribose polymerase (PARP) and inhibition of anti-apoptotic molecules like BCL2 [74].

**Fig. 9:**
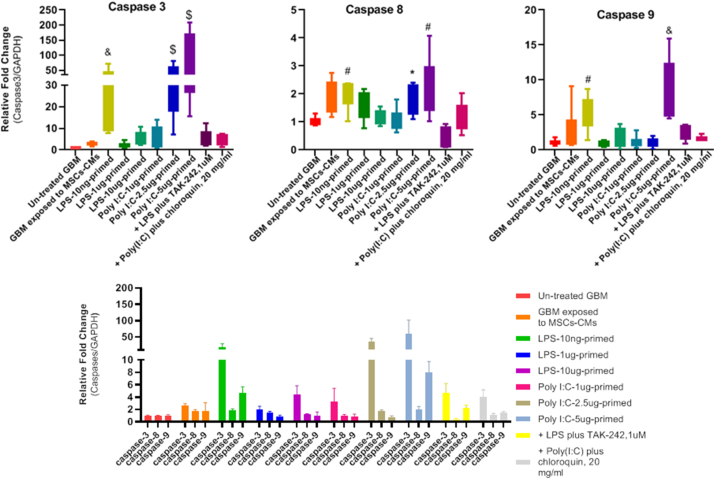
Caspase expression profiles in U87 cell lines in response to TLRs-primed-MSCs-CMs and their inhibitors treatment (*: p < 0.05, #: p < 0.01, &: p < 0.001, $: p < 0.0001).

Our result showed that pre-treatment of MSCs with Poly (I:C) significantly induces caspase 3, 8, and 9 expressions in the U87 cell line. Consistent with previous results, we observed the most overexpression levels of caspase-3 (****P value < .0001), caspase-8 (**P value < 0. 01), and −9 (***P value < .001) in 2.5 and 5 μg/ml dose of Poly (I:C). Meanwhile, TLR preconditioning drastically inhibited the expression levels of the anti-apoptotic Bcl-2 gene in all primed groups, indicating the involvement of the intrinsic pathway of apoptosis. Also, our data revealed the importance of dose-related TLR agonist effects in different groups, as we observed considerable differences in LPS (10 ng/ml) among TLR4 priming groups. Poly (I:C) pre-treatment seems to increase the mRNA levels of caspases and the production of ROS and DNA damage in the U87 cell line. It is worth mentioning that previous consistent studies reported Poly (I:C) transfection, as an innate adjuvant receptor ligand, significantly up-regulate the expression of apoptosis-associated genes and reduce the viability of all cell lines through activating both mitochondrial-mediated and extrinsic apoptotic pathways, and eventually inducing cell death [75,76]. To confirm the TLR role in cell death induction, we pretreated different groups with specific doses of TLR blockers, TAK-242, and chloroquine for TLR4 and TLR3, respectively. Interestingly, following TLR signalling pathway blockage, the expression levels of caspases considerably decreased in the mentioned groups (Fig. 9). Seemingly, the Poly (I:C) anti-tumour action partially depends on both c-Myc and cyclinD1-dependent growth arrest and caspase-dependent apoptosis, which induce anti-tumour effects and activate immune cells [77].

As mentioned above, TLR4 priming in dose 10 ng/ml considerably increased caspases-3, caspases-8, and caspase-9 mRNA expression levels in the U87 cell line. As pro-apoptotic properties of TLR3 priming have been previously demonstrated, TLR4's role in apoptosis induction is considerable [78]. TLR4 expression is still discussed controversially due to inconsistent results in both inductions of apoptosis and tumour progression following TLR4 priming. It is most likely attributed to different cell sources and differential kinetics of TLR-specific signal transduction. For instance, Sabrina Fried et al. reported activation of NF-κB and up-regulation of IFN-β and TNF-α mRNA in LPS-treated breast epithelial and cancer hybrids cell lines.

On the contrary, NF-κB phosphorylation and activation were not detected in LPS-treated parental cells. Additionally, they reported that LPS-induced cell death in hybrid cells is mainly related to overexpression of IFN-β, so IFN-β was chiefly mediated apoptosis in hybrid cells. Even though TNF-α–signalling in apoptosis cannot be ignored [79]. Based on these findings, a fusion therapy of IFN-β and tumour-targeted delivery of IFN-β may increase apoptosis by altering the mitochondrial membrane and subsequent stimulation of the caspase cascade signalling pathway.

Overall, we observed a significant increase in the apoptotic and necrotic cell population at LPS-primed-MSCs-CM and Poly (I:C)-primed-MSCs-CM group. Meanwhile, co-treatment of TAK 242-LPS, Chloroquine-Poly (I:C), BAY 11-7082-LPS, and BAY 11-7082-Poly (I:C) significantly decreased the number of apoptotic/necrotic cells. It is worth mentioning that BAY 11–7082 could not reduce early apoptotic cells and increased its cell population, which likely illustrates the independent or even inhibitor role of BAY 11–7082 in early apoptotic cell death.

Our results recommend another form of cell death, termed death receptor-induced programmed necrosis, also called necroptosis. Apoptosis is considered the immunologically quiescent form of cell death, whereas necroptosis is thought to occur following an immunological response. Necroptosis is a programmed cell death mode imitating necrosis and apoptosis features. According to previous studies, shreds of evidence demonstrated that necroptosis is induced by DRs, TLRs, interferon, and some other mediators [80].

It has been revealed that TLR-3/4 induces necroptosis in specific cells through the cascade of reactions mediated by receptor-interacting serine/threonine kinase3 (RIPK3) and MLKL. Surprisingly, our results confirmed a strong relationship between apoptotic and necrotic populations and NF-κΒ inhibitor (BAY11-7082). It significantly reduced the number of dead cells in the U87 cell line, as data obtained with flow cytometry confirmed it.

Although NF-κB is frequently considered an anti-apoptotic TF, self-regulating homeostatic balance processes, like necroptosis, seem to be associated with NF-κB [81]. NF-κB, as a TF that represent dynamic networks with various biological processes, contributes to various types of immunogenic cell deaths. According to previous studies, downstream signalling of NF-κB activation, RIPK1/RIPK3, is possibly required for the necroptotic cell's immunogenicity in the TME. RIPK1 functions as a signalling hub and coordinates activation of NF-κB following death receptors (DRs) binding by engaging vital players of the NF-κB pathway, such as NEMO and the adaptor proteins like TGF-β-activated kinase 1 binding protein 1/2 (TAB1/2) to enhance the activation of TAK1 and IκB kinase (IKK) complex [82]. As a result, NF-κB mediates the expression of pro-survival genes such as cFLIP, inhibiting caspase-8 activity and extrinsic apoptosis through creating cFLIP/caspase-8 heterodimers. However, the possibility that RIPK1 plays the opposite role (necroptotic signalling) is contingent on the kinase role of RIPK3. Due to the restricted expression of RIPK3, the intracellular dosage of this factor is requisite to determine cell death fate by apoptosis or necroptosis [83].

Along with the anti-tumour immunity of RIPK1 and RIPK3, a recent study demonstrated that exogenous injection of necroptotic cells to the TME induces RIPK-mediated secretion of cytokines. These factors promote BATF3+, DC1s, and CD8^+^ T cells' anti-tumour immunity facilitated by enhanced tumour-associated antigens presenting APCs. Notably, suppression of NF-κΒ treating BAY-117085 significantly decreased the tumour control probability, resulting in decreased immunogenicity. In this regard, NF-κB pro-inflammatory signalling downstream is a central element for the immunogenic cell death in the TME [84].

NF-κB controls the rate of multiple pro-inflammatory cytokines transcription, including TNF-α. Immunostimulants such as LPS and hypoxia can induce NF-κB translocation and TNF-α expression [85]. It is revealed that TNFα exerts multiple biological effects depending on interaction with its TNFR1 receptor. Consequently, TNFR1 binds the TNFR-associated death domain protein (TRADD) via ligation with its death domain. It recruits the receptor-interacting protein 1 (RIP1) and related components, thereby inducing the ubiquitination of RIP1 in the following steps. The mentioned reactions recruit and activate the IKK complex to induce phosphorylation of the inhibitor IκBα. It leads to its ubiquitin-mediated proteasomal degradation and facilitates its translocation. Koen MO Galenkamp et al. demonstrated that FAS is one of the primary targeted genes which NF-κB can regulate. Additionally, it appears that pathways activated by TNFR1 discard regulation of Fas expression through the NF-κB-mediated signalling pathway. TNFα feasibly induces the formation of various heterodimeric proteins of NF-κB or various post-transcription modifications, which may develop stage-specific gene regulation. It likely justifies the lack of Fas induction in various types of tumours. Epigenetic regulation, such as methylation of the Fas promoter, is another possible mechanism responsible for the TNFα failure to motivate Fas expression [86]. Additionally, IFNγ can induce the expression of Fas and caspase-8 genes in tumour cells, rendering them more susceptible to FasL treatment. The TNFα-mediated Fas overexpression is inconsistent with the standard view in which the suppression of NF-κB is considered a potential approach for cancer treatment, as several studies reported tumour-promoting potency of NF-κB. Meanwhile, NF-κB can be considered pro-apoptotic factors by stimulating pro-apoptotic proteins, including DR5, DR6, Bax, and Fas. Our results support the idea that NF-κB involved in apoptosis and, more precisely, necroptosis cell death via TNF-α-mediated Fas ligand necroptosis cell death.

**Fig. 10:**
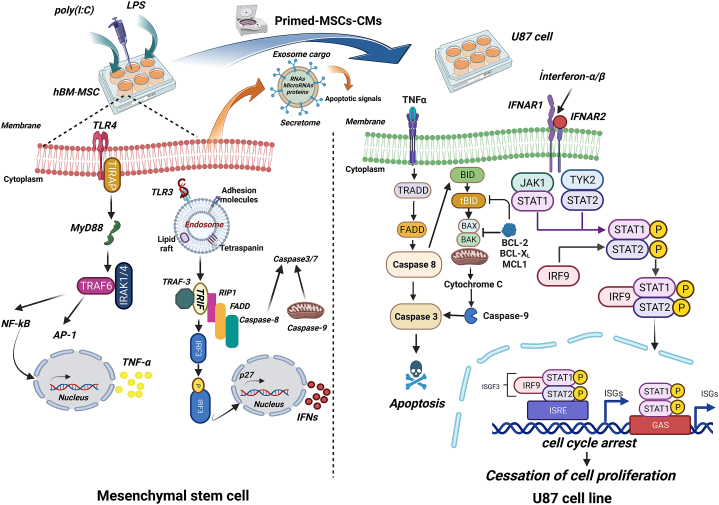
TLR signalling leads to apoptosis in tumour cells. Following TLR3 and TLR4 priming in MSCs, cascades of molecular interactions result in the secretion of various molecules in CM, known as secretome. It comprises different biomolecules, such as regulatory RNAs, miRNAs, and proteins. After removing cell derisions, CM was transferred to wells, including U87 cells. Evaluation of apoptotic markers showed us that secretome ingredients following TLRs preconditioning significantly induce apoptosis in treated u87 cell lines. The extrinsic apoptotic pathway is triggered by the ligation of TNF-α to its DRs.

Evading apoptosis in tumour cells can be bypassed by directing the necroptosis pathway and preserving anti-tumour immunity. Nevertheless, necrotic cell death may play a dual role, leading to tumour-causing inflammation and supporting all tumourigenesis stages (Fig. 10).

Consequently, the recruitment of adaptor proteins such as FADD and TRADD activates downstream factors such as Caspase-8 and 3, eventually resulting in cell apoptosis. Additionally, IFNs induce the transcription of hundreds of ISGs, which eventually induce anti-tumour effects by modulating immunomodulatory responses of tumour cells. Abbreviation: Fas-associated protein with death domain, FADD; IFN-stimulated genes, ISGs; Tumour necrosis factor receptor type 1-associated death domain protein, TRADD.

A study has ascertained that the impact of LPS treatment on viability is significantly associated with LPS concentration. Lei Lei et al. reported that exposure to higher doses of LPS induced dose-dependent reduction of cell viability. They showed that the maximum LPS concentration (10 μg/ml) causes a 52 ± 3 % reduced survival rate compared to an unprimed cell line. Similarly, 1 μg/ml of LPS reduced the cell survival rate to 64 ± 12 %. However, the results regarding 0.1 μg/ml and 0.5 μg/ml concentrations were insignificant. Moreover, higher concentrations of LPS possibly induce apoptosis in endocrine cell lines by inhibiting Bcl-2 protein expression and enhancing caspase-3 activity [87].

In previous studies, TLR priming has been a potential strategy to make critical changes in the phenotype and immunomodulatory properties of MSCs, which can enhance their therapeutic potential. It is essential to discover and understand the molecular pathways followed by TLR priming to use as an appropriate clinical application. Numerous studies have reported that TLRs-primed MSCs (LPS: 100 ng/ml and poly (I:C): 100 μg/ml) modulated neutrophil recruitment and enhanced immune cell function through the joint action of IFN-β, IL-6, and GM-CSF [88,89]. Moreover, MSC1 can recruit lymphocytes by stimulating T-cells and secreting MIP-1, CXCL9, and CCL5 [90].

Previously, it has been declared that MSC-CM can inhibit inflammation by blocking the MAPK and NF-κB signalling pathways [91]. Moreover, Jaehyup Kim et al. reported macrophages co-cultured with MSCs to activate macrophages with high-level expression of IL-10 and CD206 and low expression of IL-12, which exhibit anti-inflammatory phenotype induction and a higher phagocytic capacity [92]. Compared to unstimulated MSCs, LPS-stimulated MSCs transit to the inflammatory phase and release inflammatory mediators such as IL6 and IL1β. The link between predisposing inflammation of TLRs-primed-MSCs CM and GBM development has not been established. Pro-inflammatory compounds are divergent in regulating immune cell function, tumour growth, and anti-tumour immune responses.

Recent studies manifest that TLRs play a dual regulatory role in the tumour microenvironment (anti-tumoural and pro-tumoural effect) and offer opportunities for developing new therapeutic strategies.

Fengyuan Che et al. observed that glioma stem cells significantly stimulate the expression of inflammatory response genes (TNF-α, MIP-1α, MCP-1, IL-1β, IL-6, IL-10), cell cycle regulatory proteins (cyclin E and CDK4/6), anti-apoptotic factors (Bcl-2), and TFs (NF-κB) after activating by TLR-4 agonist LPS (0–40 μg/mL), resulting in cancer progression [93]. It has also revealed that TLR-4 activation (1 μg/ml) stimulates the secretion of the inflammatory cytokines (TNFα and ILs) and up-regulation of specific stem cell markers, including CD34 and CD133 in human glioma U251 cells [94]. The production of IL- 1β from glioma cells is responsible for the activation of TLR-4 and overexpression of HMGB1, which most likely gives rise to HLA-G, contributing to glioma immune evasion response [95,96].

Conversely, TLR3 agonists directly inhibit tumour growth by decreasing growth signals and apoptosis induction. It is supposed that TLR3 activates the extrinsic apoptosis by forming an uncommon caspase-8-activating complex comprising caspase-8/RIP1/TRIF/FADD and TLR3 [97]. TLR3 also acts directly on tumour cells through stimulation with dsRNA and promotion of tumour cell apoptosis in various tumour cells, mediating TLR3-dependent cell apoptosis [98].

Additionally, the Poly (I:C) (50 μg/mL) can suppress tumour development by induction of M1 tumour suppressor macrophages through an independent TNF-α-mediated pathway [99]. Targeting TLR3 is promising to stimulate protective anti-tumour immunity as an adjuvant therapeutic strategy. Constituent to the mentioned facts, our results showed that dysregulation of apoptotic factors, including Bcl-2, caspase-3, and caspase-9, highly depends on concentration and exposure time. Key indicators showed that suppression of Cell signalling pathways, which are involved in cellular proliferation, is closely linked to dose and time exposure. Some studies have reported that the activation of TLR3 may play a divergent role in tumour progression [100,101].

**Fig. 11:**
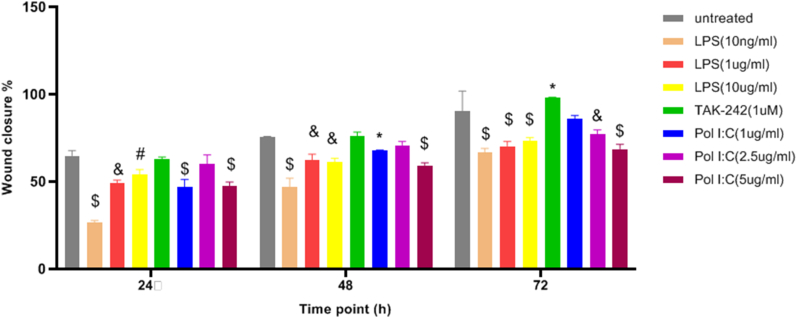
Comparison of U87 cell migration following co-culture with CMs of various TLRs and TAK-242-primed-MSCs groups. Generally, migration is decreased in TLRs-primed groups at different time points and dosages. Interestingly, the TAK-242 group did not show significant changes compared to the control group. Differences were determined over time (vs. time = 0 h) using a two-way ANOVA followed by an Uncorrected Fisher's LSD test (*: p < 0.05, #: p < 0.01, &: p < 0.001, $: p < 0.0001).

Our data suggest that TLR-priming, specifically TLR4 activation at sub-toxic dose (10 ng/ml) can disrupt GBM cells' mobility. In contrast, induced migration in the Tak-242 group confirmed the crucial role of TLR signalling in GBM migration. As discussed above, we observed attenuated migration in the TLRs-primed group. Even though TLRs priming in sub-toxic doses revealed significant variances compared to other groups (LPS: 10 ng/ml and Poly (I:C) (1 μg/ml). Fundamentally, there are conflicting results on TLR's impact on tumour cell biology. Notwithstanding the tumour-suppressing role of TLRs (and their ligands), they can induce tumour progression and enhance chemotherapy resistance [102] (Fig. 11).

Previous studies showed the divergent impact of MSCs on tumour cells, which induce both pro-tumour and anti-tumour activity, as there is a rising concern for clinical application due to their double-edged sword effects [103]. In this regard, Lu et al. reported that bone marrow MSCs could inhibit glioma U251 cell growth via suppression of the PI3K/AKT pathway [104]. Hao et al. indicated that TRAIL mediates apoptosis in glioma cells, one of the main cytotoxic agents that MSCs release [105]. Similarly, numerous studies revealed that MSCs could suppress tumour growth by blocking different signalling pathways, such as AKT and ERK1/2, in tumours such as Kaposi sarcoma and mammary carcinomas [106,107]. It has been found that MSCs can inhibit tumour cell proliferation, as a mechanistic study performed by Qiao et al. showed MSCs secrete Dickkopf-1 (Dkk-1) into the cell culture medium. This factor blocks tumour growth through Wnt signalling suppression [108]. A study by Ho et al. revealed the anti-angiogenic role of MSCs, which can be attributed to reduced expression of platelet-derived growth factor (PDGF/PDGFR), one of the well-known axes in glioma angiogenesis [109]. It is also noteworthy to mention the multifunctional role of some inflammatory factors released by MSCs, such as cytokine TGFβ; they can act as a double edge sword in cancer development, as some studies elicited the anti-tumour effect of TGFβ in various cancers [110].

On the other hand, MSCs can inhibit apoptosis and contribute to cancer pathogenesis by releasing immunosuppressive and inflammatory factors, respectively [111,112]. Additionally, numerous studies confirmed the supporting role of MSCs in tumour angiogenesis by interaction with a tumour cell and overexpression of angiogenic factors, such as TGFβ and VEGF [113].

Contradictory results are possibly attributed to different derived tissue sources, cell delivery methods, and the various dose/timing strategies of the MSC treatment [114].

## 5 Conclusion

Inflammation-related tumourigenesis has been extensively studied during previous research, but inflammation as a tumour-suppressive factor has not been fully elucidated. We have found that short-term treatment of MSCs with TLR agonists in a specific dose and time-dependent manner contributes to immunogenic cell death in the U87 cell line. On the other hand, chronic inflammation enables TME to recruit large numbers of immunosuppressive cues mediated by various cancer cells and other cell-free signals, providing a rich cancer-promoting TME and tumour resistance [115]. Owing to the critical role of inflammation in tumour biology, a deeper understanding of the linkage between inflammation and tumour biology can be essential in unravelling the hidden potential of anti-cancer treatment. So, the interplay between programmed cell death and inflammation will require further investigation to decipher whether the priming of MSCs with inflammation-relevant signals induces programmed cell death in cancer cells. It can be of future interest to improve the synergic therapeutic effects of chemo-radiation therapy. Several preclinical trials employing stem cells have been applied in cancer treatment. Considering the potent tumourigenic effects of MSCs, adjuvant PRR ligands may serve as an anti-tumour strategy and be used as a potent adjuvant for tumour vaccines or combined with conventional therapies and emerging immunotherapies. Overall, TLRs-related cell signalling pathways and innate immune inflammatory programmed cell death mediated TLRs-activated MSCs can be targeted in immune target therapy by focusing on cell-related secretomes to diminish the cancerous nature of cell-based therapy and promote anti-cancer immune responses in several ways.

## Availability of data and materials

The datasets used and analyzed during the current study are available from the corresponding author upon reasonable request.

## Ethics approval and consent to participate

The present study was approved by the Shahid Sadoughi University of Medical Sciences ethics committee (Ethics code: IR.SSU.REC.1399.090) and conducted based on previously published studies.

## Consent for publication

Not applicable.

## Funding

No funding.

## CRediT authorship contribution statement

**Seyed Mahdi Emami Meybodi:** Writing – review & editing, Writing – original draft, Visualization, Software, Resources, Project administration, Methodology, Investigation, Funding acquisition, Formal analysis, Data curation, Conceptualization. **Fateme Moradi Moraddahande:** Writing – review & editing, Writing – original draft, Visualization, Software, Resources, Project administration, Methodology, Investigation, Funding acquisition, Formal analysis, Data curation. **Ali Dehghani Firoozabadi:** Writing – review & editing, Writing – original draft, Visualization, Validation, Supervision, Software, Resources, Project administration, Methodology, Investigation, Funding acquisition, Formal analysis, Data curation, Conceptualization.

## Declaration of competing interest

The authors declare that they have no known competing financial interests or personal relationships that could have appeared to influence the work reported in this paper.
