# Supplementary material for: Immunogenic cell death mediated TLR3/4-activated MSCs in U87 GBM cell line
Source: Heliyon. 2024 Apr 22;10(9):e29858. doi: 10.1016/j.heliyon.2024.e29858 (PMC11064142; doi:10.1016/j.heliyon.2024.e29858)
Supplement: Multimedia component 2 [file mmc2.docx]

| **Interactor1** | **Category1** | **Species1** | **Interactor2** | **Category2** | **Species2** | **Score*** |
| --- | --- | --- | --- | --- | --- | --- |
| hsa-miR-16-5p | miRNA | Homo sapiens | BCL2 | mRNA | Homo sapiens | 0.8063 |
| hsa-miR-16-5p | miRNA | Homo sapiens | VEGFA | mRNA | Homo sapiens | 0.7385 |
| hsa-miR-16-5p | miRNA | Homo sapiens | CCNE1 | mRNA | Homo sapiens | 0.6685 |
| hsa-miR-16-5p | miRNA | Homo sapiens | CCND1 | mRNA | Homo sapiens | 0.6585 |
| hsa-miR-16-5p | miRNA | Homo sapiens | FGF2 | mRNA | Homo sapiens | 0.6263 |
| hsa-miR-16-5p | miRNA | Homo sapiens | Cisplatin | compound | - | 0.6146 |
| hsa-miR-16-5p | miRNA | Homo sapiens | SHOC2 | mRNA | Homo sapiens | 0.6132 |
| hsa-miR-16-5p | miRNA | Homo sapiens | Paclitaxel | compound | - | 0.6085 |
| hsa-miR-16-5p | miRNA | Homo sapiens | KIF5B | mRNA | Homo sapiens | 0.6073 |
| hsa-miR-16-5p | miRNA | Homo sapiens | E2F7 | mRNA | Homo sapiens | 0.6026 |
| hsa-miR-16-5p | miRNA | Homo sapiens | KIF23 | mRNA | Homo sapiens | 0.599 |
| hsa-miR-16-5p | miRNA | Homo sapiens | CCND2 | mRNA | Homo sapiens | 0.5971 |
| hsa-miR-16-5p | miRNA | Homo sapiens | PLAG1 | mRNA | Homo sapiens | 0.5959 |
| hsa-miR-16-5p | miRNA | Homo sapiens | CDC37L1 | mRNA | Homo sapiens | 0.5939 |
| hsa-miR-16-5p | miRNA | Homo sapiens | BMI1 | mRNA | Homo sapiens | 0.5884 |
| hsa-miR-16-5p | miRNA | Homo sapiens | PISD | mRNA | Homo sapiens | 0.5876 |
| hsa-miR-16-5p | miRNA | Homo sapiens | SMAD7 | mRNA | Homo sapiens | 0.5847 |
| hsa-miR-16-5p | miRNA | Homo sapiens | ATG9A | mRNA | Homo sapiens | 0.5838 |
| hsa-miR-16-5p | miRNA | Homo sapiens | CDCA4 | mRNA | Homo sapiens | 0.5837 |
| hsa-miR-16-5p | miRNA | Homo sapiens | SRPRA | mRNA | Homo sapiens | 0.5837 |
| hsa-miR-16-5p | miRNA | Homo sapiens | KPNA1 | mRNA | Homo sapiens | 0.5822 |
| hsa-miR-16-5p | miRNA | Homo sapiens | PURA | mRNA | Homo sapiens | 0.579 |
| hsa-miR-16-5p | miRNA | Homo sapiens | JARID2 | mRNA | Homo sapiens | 0.5787 |
| hsa-miR-16-5p | miRNA | Homo sapiens | SYPL1 | mRNA | Homo sapiens | 0.5785 |
| hsa-miR-16-5p | miRNA | Homo sapiens | MYO5A | mRNA | Homo sapiens | 0.5766 |

| Top of Form  Bottom of Form |
| --- |

| **Interactor1** | **Category1** | **Species1** | **Interactor2** | **Category2** | **Species2** | **Score*** |
| --- | --- | --- | --- | --- | --- | --- |
| hsa-miR-15a-5p | miRNA | Homo sapiens | BCL2 | mRNA | Homo sapiens | 0.7002 |
| hsa-miR-15a-5p | miRNA | Homo sapiens | VEGFA | mRNA | Homo sapiens | 0.693 |
| hsa-miR-15a-5p | miRNA | Homo sapiens | CCND1 | mRNA | Homo sapiens | 0.6379 |
| hsa-miR-15a-5p | miRNA | Homo sapiens | CCNE1 | mRNA | Homo sapiens | 0.6294 |
| hsa-miR-15a-5p | miRNA | Homo sapiens | YAP1 | mRNA | Homo sapiens | 0.6185 |
| hsa-miR-15a-5p | miRNA | Homo sapiens | Cisplatin | compound | - | 0.603 |
| hsa-miR-15a-5p | miRNA | Homo sapiens | E2F7 | mRNA | Homo sapiens | 0.6026 |
| hsa-miR-15a-5p | miRNA | Homo sapiens | SHOC2 | mRNA | Homo sapiens | 0.6019 |
| hsa-miR-15a-5p | miRNA | Homo sapiens | CCND2 | mRNA | Homo sapiens | 0.6011 |
| hsa-miR-15a-5p | miRNA | Homo sapiens | PLAG1 | mRNA | Homo sapiens | 0.5959 |
| hsa-miR-15a-5p | miRNA | Homo sapiens | CDC37L1 | mRNA | Homo sapiens | 0.5939 |
| hsa-miR-15a-5p | miRNA | Homo sapiens | Gemcitabine | compound | - | 0.5882 |
| hsa-miR-15a-5p | miRNA | Homo sapiens | KIF23 | mRNA | Homo sapiens | 0.587 |
| hsa-miR-15a-5p | miRNA | Homo sapiens | CDCA4 | mRNA | Homo sapiens | 0.5837 |
| hsa-miR-15a-5p | miRNA | Homo sapiens | KIF5B | mRNA | Homo sapiens | 0.5837 |
| hsa-miR-15a-5p | miRNA | Homo sapiens | JARID2 | mRNA | Homo sapiens | 0.5787 |
| hsa-miR-15a-5p | miRNA | Homo sapiens | MYO5A | mRNA | Homo sapiens | 0.5766 |
| hsa-miR-15a-5p | miRNA | Homo sapiens | SMAD7 | mRNA | Homo sapiens | 0.5763 |
| hsa-miR-15a-5p | miRNA | Homo sapiens | MAFK | mRNA | Homo sapiens | 0.5746 |
| hsa-miR-15a-5p | miRNA | Homo sapiens | PISD | mRNA | Homo sapiens | 0.5743 |
| hsa-miR-15a-5p | miRNA | Homo sapiens | ZNF367 | mRNA | Homo sapiens | 0.5743 |
| hsa-miR-15a-5p | miRNA | Homo sapiens | UBN2 | mRNA | Homo sapiens | 0.5727 |
| hsa-miR-15a-5p | miRNA | Homo sapiens | ATG9A | mRNA | Homo sapiens | 0.5712 |
| hsa-miR-15a-5p | miRNA | Homo sapiens | SRPRA | mRNA | Homo sapiens | 0.5711 |
| hsa-miR-15a-5p | miRNA | Homo sapiens | HOXA3 | mRNA | Homo sapiens | 0.571 |

| **Interactor1** | **Category1** | **Species1** | **Interactor2** | **Category2** | **Species2** | **Score*** |
| --- | --- | --- | --- | --- | --- | --- |
| hsa-miR-7641 | miRNA | Homo sapiens | BANK1 | protein | Homo sapiens | 0.5448 |
| hsa-miR-7641 | miRNA | Homo sapiens | TRIP4 | protein | Homo sapiens | 0.5342 |
| hsa-miR-7641 | miRNA | Homo sapiens | TAOK1 | protein | Homo sapiens | 0.5298 |
| hsa-miR-7641 | miRNA | Homo sapiens | BANK1 | mRNA | Homo sapiens | 0.5103 |
| hsa-miR-7641 | miRNA | Homo sapiens | TRIP4 | mRNA | Homo sapiens | 0.5103 |
| hsa-miR-7641 | miRNA | Homo sapiens | ARL5C | protein | Homo sapiens | 0.4938 |
| hsa-miR-7641 | miRNA | Homo sapiens | TAOK1 | mRNA | Homo sapiens | 0.4913 |
| hsa-miR-7641 | miRNA | Homo sapiens | ARL5C | mRNA | Homo sapiens | 0.4621 |
| hsa-miR-7641 | miRNA | Homo sapiens | ATIC | mRNA | Homo sapiens | 0.461 |
| hsa-miR-7641 | miRNA | Homo sapiens | ACAA1 | mRNA | Homo sapiens | 0.4348 |
| hsa-miR-7641 | miRNA | Homo sapiens | COX20 | mRNA | Homo sapiens | 0.4195 |
| hsa-miR-7641 | miRNA | Homo sapiens | COX20 | protein | Homo sapiens | 0.4195 |
| hsa-miR-7641 | miRNA | Homo sapiens | CDKN2A | mRNA | Homo sapiens | 0.4073 |
| hsa-miR-7641 | miRNA | Homo sapiens | REL | protein | Homo sapiens | 0.394 |
| hsa-miR-7641 | miRNA | Homo sapiens | ETNK1 | mRNA | Homo sapiens | 0.3733 |
| hsa-miR-7641 | miRNA | Homo sapiens | PPWD1 | protein | Homo sapiens | 0.3724 |
| hsa-miR-7641 | miRNA | Homo sapiens | PPWD1 | mRNA | Homo sapiens | 0.3724 |
| hsa-miR-7641 | miRNA | Homo sapiens | SLC30A4 | protein | Homo sapiens | 0.3675 |
| hsa-miR-7641 | miRNA | Homo sapiens | SLC30A4 | mRNA | Homo sapiens | 0.3675 |
| hsa-miR-7641 | miRNA | Homo sapiens | REL | mRNA | Homo sapiens | 0.3663 |
| hsa-miR-7641 | miRNA | Homo sapiens | RTN3 | mRNA | Homo sapiens | 0.3647 |
| hsa-miR-7641 | miRNA | Homo sapiens | SCAI | mRNA | Homo sapiens | 0.3534 |
| hsa-miR-7641 | miRNA | Homo sapiens | TFAP2A | mRNA | Homo sapiens | 0.3534 |
| hsa-miR-7641 | miRNA | Homo sapiens | MSC | mRNA | Homo sapiens | 0.3426 |
| hsa-miR-7641 | miRNA | Homo sapiens | SIX1 | TF | Homo sapiens | 0.3383 |

|  |
| --- |

| **Interactor1** | **Category1** | **Species1** | **Interactor2** | **Category2** | **Species2** | **Score*** |
| --- | --- | --- | --- | --- | --- | --- |
| hsa-miR-142-5p | miRNA | Homo sapiens | Doxorubicin | compound | - | 0.6256 |
| hsa-miR-142-5p | miRNA | Homo sapiens | Topotecan | compound | - | 0.5931 |
| hsa-miR-142-5p | miRNA | Homo sapiens | CCND1 | mRNA | Homo sapiens | 0.5608 |
| hsa-miR-142-5p | miRNA | Homo sapiens | LAPTM4A | mRNA | Homo sapiens | 0.5608 |
| hsa-miR-142-5p | miRNA | Homo sapiens | FEM1C | mRNA | Homo sapiens | 0.5514 |
| hsa-miR-142-5p | miRNA | Homo sapiens | ACTN4 | mRNA | Homo sapiens | 0.5419 |
| hsa-miR-142-5p | miRNA | Homo sapiens | PCBP1 | mRNA | Homo sapiens | 0.5379 |
| hsa-miR-142-5p | miRNA | Homo sapiens | AKAP11 | mRNA | Homo sapiens | 0.5377 |
| hsa-miR-142-5p | miRNA | Homo sapiens | XIAP | mRNA | Homo sapiens | 0.5366 |
| hsa-miR-142-5p | miRNA | Homo sapiens | HMGB1 | mRNA | Homo sapiens | 0.5354 |
| hsa-miR-142-5p | miRNA | Homo sapiens | CHAC1 | mRNA | Homo sapiens | 0.5266 |
| hsa-miR-142-5p | miRNA | Homo sapiens | B2M | mRNA | Homo sapiens | 0.5249 |
| hsa-miR-142-5p | miRNA | Homo sapiens | CAPRIN2 | mRNA | Homo sapiens | 0.5228 |
| hsa-miR-142-5p | miRNA | Homo sapiens | PTP4A1 | mRNA | Homo sapiens | 0.5219 |
| hsa-miR-142-5p | miRNA | Homo sapiens | ZFYVE26 | mRNA | Homo sapiens | 0.5188 |
| hsa-miR-142-5p | miRNA | Homo sapiens | DUSP2 | mRNA | Homo sapiens | 0.5188 |
| hsa-miR-142-5p | miRNA | Homo sapiens | ZFYVE21 | mRNA | Homo sapiens | 0.5147 |
| hsa-miR-142-5p | miRNA | Homo sapiens | PTPN4 | mRNA | Homo sapiens | 0.5126 |
| hsa-miR-142-5p | miRNA | Homo sapiens | ZBTB43 | mRNA | Homo sapiens | 0.5121 |
| hsa-miR-142-5p | miRNA | Homo sapiens | CEP97 | mRNA | Homo sapiens | 0.5121 |
| hsa-miR-142-5p | miRNA | Homo sapiens | EGLN3 | mRNA | Homo sapiens | 0.5114 |
| hsa-miR-142-5p | miRNA | Homo sapiens | MED17 | mRNA | Homo sapiens | 0.5114 |
| hsa-miR-142-5p | miRNA | Homo sapiens | SAMD12 | mRNA | Homo sapiens | 0.5114 |
| hsa-miR-142-5p | miRNA | Homo sapiens | NFE2L2 | mRNA | Homo sapiens | 0.5104 |
| hsa-miR-142-5p | miRNA | Homo sapiens | ETNK1 | mRNA | Homo sapiens | 0.5083 |

| **Interactor1** | **Category1** | **Species1** | **Interactor2** | **Category2** | **Species2** | **Score*** |
| --- | --- | --- | --- | --- | --- | --- |
| hsa-miR-1285-3p | miRNA | Homo sapiens | JUN | mRNA | Homo sapiens | 0.481 |
| hsa-miR-1285-3p | miRNA | Homo sapiens | GNAI2 | mRNA | Homo sapiens | 0.4534 |
| hsa-miR-1285-3p | miRNA | Homo sapiens | E2F7 | mRNA | Homo sapiens | 0.4457 |
| hsa-miR-1285-3p | miRNA | Homo sapiens | PTAR1 | mRNA | Homo sapiens | 0.4457 |
| hsa-miR-1285-3p | miRNA | Homo sapiens | ACTR2 | mRNA | Homo sapiens | 0.4457 |
| hsa-miR-1285-3p | miRNA | Homo sapiens | CEP250 | mRNA | Homo sapiens | 0.4457 |
| hsa-miR-1285-3p | miRNA | Homo sapiens | HNRNPD | mRNA | Homo sapiens | 0.4409 |
| hsa-miR-1285-3p | miRNA | Homo sapiens | TSC22D3 | mRNA | Homo sapiens | 0.4392 |
| hsa-miR-1285-3p | miRNA | Homo sapiens | SEMA4C | mRNA | Homo sapiens | 0.424 |
| hsa-miR-1285-3p | miRNA | Homo sapiens | UBB | mRNA | Homo sapiens | 0.4201 |
| hsa-miR-1285-3p | miRNA | Homo sapiens | Gemcitabine | compound | - | 0.4197 |
| hsa-miR-1285-3p | miRNA | Homo sapiens | BTRC | mRNA | Homo sapiens | 0.4191 |
| hsa-miR-1285-3p | miRNA | Homo sapiens | TP53 | mRNA | Homo sapiens | 0.4166 |
| hsa-miR-1285-3p | miRNA | Homo sapiens | SRPRA | mRNA | Homo sapiens | 0.4144 |
| hsa-miR-1285-3p | miRNA | Homo sapiens | Eloxatine | compound | - | 0.4114 |
| hsa-miR-1285-3p | miRNA | Homo sapiens | FUT1 | protein | Homo sapiens | 0.4021 |
| hsa-miR-1285-3p | miRNA | Homo sapiens | UGDH | mRNA | Homo sapiens | 0.4021 |
| hsa-miR-1285-3p | miRNA | Homo sapiens | TMEM220 | mRNA | Homo sapiens | 0.3998 |
| hsa-miR-1285-3p | miRNA | Homo sapiens | TOMM5 | mRNA | Homo sapiens | 0.3982 |
| hsa-miR-1285-3p | miRNA | Homo sapiens | PAFAH1B1 | mRNA | Homo sapiens | 0.3928 |
| hsa-miR-1285-3p | miRNA | Homo sapiens | UGDH | protein | Homo sapiens | 0.3916 |
| hsa-miR-1285-3p | miRNA | Homo sapiens | RAB34 | mRNA | Homo sapiens | 0.3854 |
| hsa-miR-1285-3p | miRNA | Homo sapiens | ZBTB34 | protein | Homo sapiens | 0.3834 |
| hsa-miR-1285-3p | miRNA | Homo sapiens | MT1E | mRNA | Homo sapiens | 0.3822 |
| hsa-miR-1285-3p | miRNA | Homo sapiens | PWP1 | mRNA | Homo sapiens | 0.3822 |

| **Interactor1** | **Category1** | **Species1** | **Interactor2** | **Category2** | **Species2** | **Score*** |
| --- | --- | --- | --- | --- | --- | --- |
| hsa-miR-22-3p | miRNA | Homo sapiens | Cisplatin | compound | - | 0.6234 |
| hsa-miR-22-3p | miRNA | Homo sapiens | YWHAZ | mRNA | Homo sapiens | 0.6217 |
| hsa-miR-22-3p | miRNA | Homo sapiens | RAB5B | mRNA | Homo sapiens | 0.6007 |
| hsa-miR-22-3p | miRNA | Homo sapiens | SIRT1 | mRNA | Homo sapiens | 0.6007 |
| hsa-miR-22-3p | miRNA | Homo sapiens | C5orf24 | mRNA | Homo sapiens | 0.5977 |
| hsa-miR-22-3p | miRNA | Homo sapiens | PTEN | mRNA | Homo sapiens | 0.5941 |
| hsa-miR-22-3p | miRNA | Homo sapiens | SP1 | mRNA | Homo sapiens | 0.5768 |
| hsa-miR-22-3p | miRNA | Homo sapiens | SNAI1 | mRNA | Homo sapiens | 0.5694 |
| hsa-miR-22-3p | miRNA | Homo sapiens | 5-Fluorouracil | compound | - | 0.5691 |
| hsa-miR-22-3p | miRNA | Homo sapiens | FTL | mRNA | Homo sapiens | 0.5677 |
| hsa-miR-22-3p | miRNA | Homo sapiens | Doxorubicin | compound | - | 0.5556 |
| hsa-miR-22-3p | miRNA | Homo sapiens | PAFAH1B2 | mRNA | Homo sapiens | 0.5487 |
| hsa-miR-22-3p | miRNA | Homo sapiens | MDC1 | mRNA | Homo sapiens | 0.5468 |
| hsa-miR-22-3p | miRNA | Homo sapiens | LRRC1 | mRNA | Homo sapiens | 0.5424 |
| hsa-miR-22-3p | miRNA | Homo sapiens | CHD9 | mRNA | Homo sapiens | 0.5422 |
| hsa-miR-22-3p | miRNA | Homo sapiens | ACLY | mRNA | Homo sapiens | 0.5421 |
| hsa-miR-22-3p | miRNA | Homo sapiens | TIAM1 | mRNA | Homo sapiens | 0.5414 |
| hsa-miR-22-3p | miRNA | Homo sapiens | ERBB3 | mRNA | Homo sapiens | 0.5381 |
| hsa-miR-22-3p | miRNA | Homo sapiens | BTF3 | mRNA | Homo sapiens | 0.5352 |
| hsa-miR-22-3p | miRNA | Homo sapiens | Paclitaxel | compound | - | 0.5261 |
| hsa-miR-22-3p | miRNA | Homo sapiens | ESR1 | mRNA | Homo sapiens | 0.525 |
| hsa-miR-22-3p | miRNA | Homo sapiens | VSNL1 | mRNA | Homo sapiens | 0.5244 |
| hsa-miR-22-3p | miRNA | Homo sapiens | TET2 | mRNA | Homo sapiens | 0.5244 |
| hsa-miR-22-3p | miRNA | Homo sapiens | RBMS1 | mRNA | Homo sapiens | 0.5228 |
| hsa-miR-22-3p | miRNA | Homo sapiens | BRWD3 | mRNA | Homo sapiens | 0.5226 |

| **Interactor1** | **Category1** | **Species1** | **Interactor2** | **Category2** | **Species2** | **Score*** |
| --- | --- | --- | --- | --- | --- | --- |
| hsa-miR-181c-5p | miRNA | Homo sapiens | KRAS | mRNA | Homo sapiens | 0.5872 |
| hsa-miR-181c-5p | miRNA | Homo sapiens | BCL2 | mRNA | Homo sapiens | 0.5596 |
| hsa-miR-181c-5p | miRNA | Homo sapiens | RLF | mRNA | Homo sapiens | 0.5493 |
| hsa-miR-181c-5p | miRNA | Homo sapiens | ZFP36L1 | mRNA | Homo sapiens | 0.5493 |
| hsa-miR-181c-5p | miRNA | Homo sapiens | GSKIP | mRNA | Homo sapiens | 0.5493 |
| hsa-miR-181c-5p | miRNA | Homo sapiens | DDX3X | mRNA | Homo sapiens | 0.5466 |
| hsa-miR-181c-5p | miRNA | Homo sapiens | CARM1 | mRNA | Homo sapiens | 0.5463 |
| hsa-miR-181c-5p | miRNA | Homo sapiens | Cisplatin | compound | - | 0.5378 |
| hsa-miR-181c-5p | miRNA | Homo sapiens | TNPO1 | mRNA | Homo sapiens | 0.5354 |
| hsa-miR-181c-5p | miRNA | Homo sapiens | DDIT4 | mRNA | Homo sapiens | 0.5265 |
| hsa-miR-181c-5p | miRNA | Homo sapiens | AFTPH | mRNA | Homo sapiens | 0.5249 |
| hsa-miR-181c-5p | miRNA | Homo sapiens | BRD1 | mRNA | Homo sapiens | 0.5249 |
| hsa-miR-181c-5p | miRNA | Homo sapiens | ZFAND6 | mRNA | Homo sapiens | 0.5241 |
| hsa-miR-181c-5p | miRNA | Homo sapiens | ATM | mRNA | Homo sapiens | 0.5156 |
| hsa-miR-181c-5p | miRNA | Homo sapiens | ZEB2 | mRNA | Homo sapiens | 0.5143 |
| hsa-miR-181c-5p | miRNA | Homo sapiens | CPT1A | mRNA | Homo sapiens | 0.5139 |
| hsa-miR-181c-5p | miRNA | Homo sapiens | GLCCI1 | mRNA | Homo sapiens | 0.5121 |
| hsa-miR-181c-5p | miRNA | Homo sapiens | TGIF2 | mRNA | Homo sapiens | 0.5121 |
| hsa-miR-181c-5p | miRNA | Homo sapiens | MTPN | mRNA | Homo sapiens | 0.5121 |
| hsa-miR-181c-5p | miRNA | Homo sapiens | TMEM64 | mRNA | Homo sapiens | 0.5121 |
| hsa-miR-181c-5p | miRNA | Homo sapiens | SPTY2D1 | mRNA | Homo sapiens | 0.5121 |
| hsa-miR-181c-5p | miRNA | Homo sapiens | ZBTB43 | mRNA | Homo sapiens | 0.5121 |
| hsa-miR-181c-5p | miRNA | Homo sapiens | SRSF7 | mRNA | Homo sapiens | 0.5121 |
| hsa-miR-181c-5p | miRNA | Homo sapiens | CBX4 | mRNA | Homo sapiens | 0.5071 |
| hsa-miR-181c-5p | miRNA | Homo sapiens | PRKCD | mRNA | Homo sapiens | 0.5021 |

| **Interactor1** | **Category1** | **Species1** | **Interactor2** | **Category2** | **Species2** | **Score*** |
| --- | --- | --- | --- | --- | --- | --- |
| hsa-miR-10a-5p | miRNA | Homo sapiens | H3F3B | mRNA | Homo sapiens | 0.716 |
| hsa-miR-10a-5p | miRNA | Homo sapiens | BCL2L11 | mRNA | Homo sapiens | 0.7156 |
| hsa-miR-10a-5p | miRNA | Homo sapiens | MIR140 | miRNA | Homo sapiens | 0.686 |
| hsa-miR-10a-5p | miRNA | Homo sapiens | Cisplatin | compound | - | 0.685 |
| hsa-miR-10a-5p | miRNA | Homo sapiens | PUM2 | mRNA | Homo sapiens | 0.6762 |
| hsa-miR-10a-5p | miRNA | Homo sapiens | RORA | mRNA | Homo sapiens | 0.6718 |
| hsa-miR-10a-5p | miRNA | Homo sapiens | RBM12B | mRNA | Homo sapiens | 0.6684 |
| hsa-miR-10a-5p | miRNA | Homo sapiens | WEE1 | mRNA | Homo sapiens | 0.6632 |
| hsa-miR-10a-5p | miRNA | Homo sapiens | CDK6 | mRNA | Homo sapiens | 0.6595 |
| hsa-miR-10a-5p | miRNA | Homo sapiens | ACTG1 | mRNA | Homo sapiens | 0.6489 |
| hsa-miR-10a-5p | miRNA | Homo sapiens | BCL6 | mRNA | Homo sapiens | 0.644 |
| hsa-miR-10a-5p | miRNA | Homo sapiens | E2F7 | mRNA | Homo sapiens | 0.6438 |
| hsa-miR-10a-5p | miRNA | Homo sapiens | EIF1 | mRNA | Homo sapiens | 0.6434 |
| hsa-miR-10a-5p | miRNA | Homo sapiens | MKNK2 | mRNA | Homo sapiens | 0.6434 |
| hsa-miR-10a-5p | miRNA | Homo sapiens | ZNF367 | mRNA | Homo sapiens | 0.6429 |
| hsa-miR-10a-5p | miRNA | Homo sapiens | RAB21 | mRNA | Homo sapiens | 0.6408 |
| hsa-miR-10a-5p | miRNA | Homo sapiens | TP53INP1 | mRNA | Homo sapiens | 0.6394 |
| hsa-miR-10a-5p | miRNA | Homo sapiens | AKAP11 | mRNA | Homo sapiens | 0.6387 |
| hsa-miR-10a-5p | miRNA | Homo sapiens | ARHGAP12 | mRNA | Homo sapiens | 0.6373 |
| hsa-miR-10a-5p | miRNA | Homo sapiens | BNIP2 | mRNA | Homo sapiens | 0.6347 |
| hsa-miR-10a-5p | miRNA | Homo sapiens | GPCPD1 | mRNA | Homo sapiens | 0.6315 |
| hsa-miR-10a-5p | miRNA | Homo sapiens | TPM4 | mRNA | Homo sapiens | 0.6311 |
| hsa-miR-10a-5p | miRNA | Homo sapiens | SNX4 | mRNA | Homo sapiens | 0.631 |
| hsa-miR-10a-5p | miRNA | Homo sapiens | CELF1 | mRNA | Homo sapiens | 0.6276 |
| hsa-miR-10a-5p | miRNA | Homo sapiens | PAFAH1B1 | mRNA | Homo sapiens | 0.625 |

| **Interactor1** | **Category1** | **Species1** | **Interactor2** | **Category2** | **Species2** | **Score*** |
| --- | --- | --- | --- | --- | --- | --- |
| hsa-miR-151a-3p | miRNA | Homo sapiens | HIF1A | mRNA | Homo sapiens | 0.5637 |
| hsa-miR-151a-3p | miRNA | Homo sapiens | BRWD1 | mRNA | Homo sapiens | 0.5387 |
| hsa-miR-151a-3p | miRNA | Homo sapiens | SEPT8 | mRNA | Homo sapiens | 0.5379 |
| hsa-miR-151a-3p | miRNA | Homo sapiens | SEPT8 | protein | Homo sapiens | 0.5 |
| hsa-miR-151a-3p | miRNA | Homo sapiens | MCL1 | mRNA | Homo sapiens | 0.4995 |
| hsa-miR-151a-3p | miRNA | Homo sapiens | ZBTB24 | mRNA | Homo sapiens | 0.4994 |
| hsa-miR-151a-3p | miRNA | Homo sapiens | CREBRF | mRNA | Homo sapiens | 0.4825 |
| hsa-miR-151a-3p | miRNA | Homo sapiens | Gemcitabine | compound | - | 0.4721 |
| hsa-miR-151a-3p | miRNA | Homo sapiens | EFNA3 | mRNA | Homo sapiens | 0.4462 |
| hsa-miR-151a-3p | miRNA | Homo sapiens | BIRC3 | mRNA | Homo sapiens | 0.4457 |
| hsa-miR-151a-3p | miRNA | Homo sapiens | GNAI3 | mRNA | Homo sapiens | 0.4457 |
| hsa-miR-151a-3p | miRNA | Homo sapiens | UFL1 | mRNA | Homo sapiens | 0.4457 |
| hsa-miR-151a-3p | miRNA | Homo sapiens | MORF4L2 | mRNA | Homo sapiens | 0.4435 |
| hsa-miR-151a-3p | miRNA | Homo sapiens | NME7 | mRNA | Homo sapiens | 0.4332 |
| EIF4G2 | lncRNA | Homo sapiens | hsa-miR-151a-3p | miRNA | Homo sapiens | 0.4262 |
| hsa-miR-151a-3p | miRNA | Homo sapiens | Docetaxel | compound | - | 0.4235 |
| hsa-miR-151a-3p | miRNA | Homo sapiens | INTU | mRNA | Homo sapiens | 0.4195 |
| hsa-miR-151a-3p | miRNA | Homo sapiens | INTU | protein | Homo sapiens | 0.4195 |
| hsa-miR-151a-3p | miRNA | Homo sapiens | KBTBD2 | mRNA | Homo sapiens | 0.419 |
| hsa-miR-151a-3p | miRNA | Homo sapiens | PURB | mRNA | Homo sapiens | 0.4189 |
| hsa-miR-151a-3p | miRNA | Homo sapiens | UCHL1 | mRNA | Homo sapiens | 0.4144 |
| hsa-miR-151a-3p | miRNA | Homo sapiens | RYBP | mRNA | Homo sapiens | 0.4143 |
| hsa-miR-151a-3p | miRNA | Homo sapiens | ACTR2 | mRNA | Homo sapiens | 0.4132 |
| hsa-miR-151a-3p | miRNA | Homo sapiens | MCL1 | protein | Homo sapiens | 0.4118 |
| hsa-miR-151a-3p | miRNA | Homo sapiens | Eloxatine | compound | - | 0.4114 |

| **Interactor1** | **Category1** | **Species1** | **Interactor2** | **Category2** | **Species2** | **Score*** |
| --- | --- | --- | --- | --- | --- | --- |
| hsa-miR-19a-3p | miRNA | Homo sapiens | PTEN | mRNA | Homo sapiens | 0.7548 |
| hsa-miR-19a-3p | miRNA | Homo sapiens | ZNF800 | mRNA | Homo sapiens | 0.6369 |
| hsa-miR-19a-3p | miRNA | Homo sapiens | PPP1R15B | mRNA | Homo sapiens | 0.6294 |
| hsa-miR-19a-3p | miRNA | Homo sapiens | MSMO1 | mRNA | Homo sapiens | 0.6271 |
| hsa-miR-19a-3p | miRNA | Homo sapiens | ZBTB4 | mRNA | Homo sapiens | 0.6269 |
| hsa-miR-19a-3p | miRNA | Homo sapiens | SOCS1 | mRNA | Homo sapiens | 0.6249 |
| hsa-miR-19a-3p | miRNA | Homo sapiens | CLIP1 | mRNA | Homo sapiens | 0.6248 |
| hsa-miR-19a-3p | miRNA | Homo sapiens | CREBL2 | mRNA | Homo sapiens | 0.6191 |
| hsa-miR-19a-3p | miRNA | Homo sapiens | CD164 | mRNA | Homo sapiens | 0.6187 |
| hsa-miR-19a-3p | miRNA | Homo sapiens | MAPK1 | mRNA | Homo sapiens | 0.6181 |
| hsa-miR-19a-3p | miRNA | Homo sapiens | C16orf70 | mRNA | Homo sapiens | 0.6151 |
| hsa-miR-19a-3p | miRNA | Homo sapiens | RHOB | mRNA | Homo sapiens | 0.615 |
| hsa-miR-19a-3p | miRNA | Homo sapiens | SERINC3 | mRNA | Homo sapiens | 0.6139 |
| hsa-miR-19a-3p | miRNA | Homo sapiens | RAPGEF2 | mRNA | Homo sapiens | 0.6122 |
| hsa-miR-19a-3p | miRNA | Homo sapiens | NPTN | mRNA | Homo sapiens | 0.6122 |
| hsa-miR-19a-3p | miRNA | Homo sapiens | ARHGAP1 | mRNA | Homo sapiens | 0.6115 |
| hsa-miR-19a-3p | miRNA | Homo sapiens | RAB14 | mRNA | Homo sapiens | 0.6098 |
| hsa-miR-19a-3p | miRNA | Homo sapiens | SOX4 | mRNA | Homo sapiens | 0.6095 |
| hsa-miR-19a-3p | miRNA | Homo sapiens | ARL8A | mRNA | Homo sapiens | 0.6081 |
| hsa-miR-19a-3p | miRNA | Homo sapiens | SMAD4 | mRNA | Homo sapiens | 0.606 |
| hsa-miR-19a-3p | miRNA | Homo sapiens | RAB5B | mRNA | Homo sapiens | 0.6055 |
| hsa-miR-19a-3p | miRNA | Homo sapiens | TNRC6A | mRNA | Homo sapiens | 0.6026 |
| hsa-miR-19a-3p | miRNA | Homo sapiens | BLCAP | mRNA | Homo sapiens | 0.6019 |
| hsa-miR-19a-3p | miRNA | Homo sapiens | H19 | lncRNA | Homo sapiens | 0.6009 |
| hsa-miR-19a-3p | miRNA | Homo sapiens | TGFBR2 | mRNA | Homo sapiens | 0.6005 |

| **Interactor1** | **Category1** | **Species1** | **Interactor2** | **Category2** | **Species2** | **Score*** |
| --- | --- | --- | --- | --- | --- | --- |
| hsa-miR-877-5p | miRNA | Homo sapiens | PCNA | mRNA | Homo sapiens | 0.4938 |
| hsa-miR-877-5p | miRNA | Homo sapiens | CDKN1B | mRNA | Homo sapiens | 0.4715 |
| hsa-miR-877-5p | miRNA | Homo sapiens | UBN2 | mRNA | Homo sapiens | 0.4673 |
| hsa-miR-877-5p | miRNA | Homo sapiens | EEF2K | mRNA | Homo sapiens | 0.4656 |
| hsa-miR-877-5p | miRNA | Homo sapiens | BTG2 | mRNA | Homo sapiens | 0.4624 |
| hsa-miR-877-5p | miRNA | Homo sapiens | LNPEP | mRNA | Homo sapiens | 0.4564 |
| hsa-miR-877-5p | miRNA | Homo sapiens | UBE2N | mRNA | Homo sapiens | 0.4564 |
| hsa-miR-877-5p | miRNA | Homo sapiens | PBRM1 | mRNA | Homo sapiens | 0.4564 |
| hsa-miR-877-5p | miRNA | Homo sapiens | GOLGA1 | mRNA | Homo sapiens | 0.4528 |
| hsa-miR-877-5p | miRNA | Homo sapiens | WWTR1 | mRNA | Homo sapiens | 0.4528 |
| hsa-miR-877-5p | miRNA | Homo sapiens | MAN1A1 | mRNA | Homo sapiens | 0.4528 |
| hsa-miR-877-5p | miRNA | Homo sapiens | XPO7 | mRNA | Homo sapiens | 0.4528 |
| hsa-miR-877-5p | miRNA | Homo sapiens | CAP1 | mRNA | Homo sapiens | 0.4528 |
| hsa-miR-877-5p | miRNA | Homo sapiens | POU2F1 | mRNA | Homo sapiens | 0.4528 |
| hsa-miR-877-5p | miRNA | Homo sapiens | ZNF318 | mRNA | Homo sapiens | 0.4528 |
| hsa-miR-877-5p | miRNA | Homo sapiens | CREBRF | mRNA | Homo sapiens | 0.4528 |
| hsa-miR-877-5p | miRNA | Homo sapiens | SERPINB9 | mRNA | Homo sapiens | 0.4528 |
| hsa-miR-877-5p | miRNA | Homo sapiens | NCBP2 | mRNA | Homo sapiens | 0.4525 |
| hsa-miR-877-5p | miRNA | Homo sapiens | SRSF3 | mRNA | Homo sapiens | 0.4525 |
| hsa-miR-877-5p | miRNA | Homo sapiens | AFF4 | mRNA | Homo sapiens | 0.4457 |
| hsa-miR-877-5p | miRNA | Homo sapiens | ARL6IP1 | mRNA | Homo sapiens | 0.4457 |
| hsa-miR-877-5p | miRNA | Homo sapiens | B3GNT7 | mRNA | Homo sapiens | 0.4457 |
| hsa-miR-877-5p | miRNA | Homo sapiens | CD46 | mRNA | Homo sapiens | 0.4457 |
| hsa-miR-877-5p | miRNA | Homo sapiens | CYBRD1 | mRNA | Homo sapiens | 0.4457 |
| hsa-miR-877-5p | miRNA | Homo sapiens | EPRS | mRNA | Homo sapiens | 0.4457 |

| **mir NAME** | **log2FoldChange** | **pvalue** |
| --- | --- | --- |
| hsa-miR-16-5p | 1.266964012 | 8.03E-07 |
| hsa-miR-15a-5p | 1.175241057 | 0.000578531 |
| hsa-miR-7641 | -2.261743359 | 0.000601834 |
| hsa-miR-142-5p | 1.186835731 | 0.001045163 |
| hsa-miR-1285-3p | 1.709983061 | 0.002643878 |
| hsa-miR-22-3p | 1.243466822 | 0.003112665 |
| hsa-miR-181c-5p | 1.167670479 | 0.005876021 |
| hsa-miR-10a-5p | -0.922777207 | 0.00593462 |
| hsa-miR-151a-3p | -0.789381392 | 0.006145565 |
| hsa-miR-19a-3p | -1.75792462 | 0.007861902 |
| hsa-miR-877-5p | 1.971426571 | 0.009188833 |
