# Supplementary figures and images for: Immunogenic cell death mediated TLR3/4-activated MSCs in U87 GBM cell line
Source: Heliyon. 2024 Apr 22;10(9):e29858. doi: 10.1016/j.heliyon.2024.e29858 (PMC11064142; doi:10.1016/j.heliyon.2024.e29858)

## Slide 1
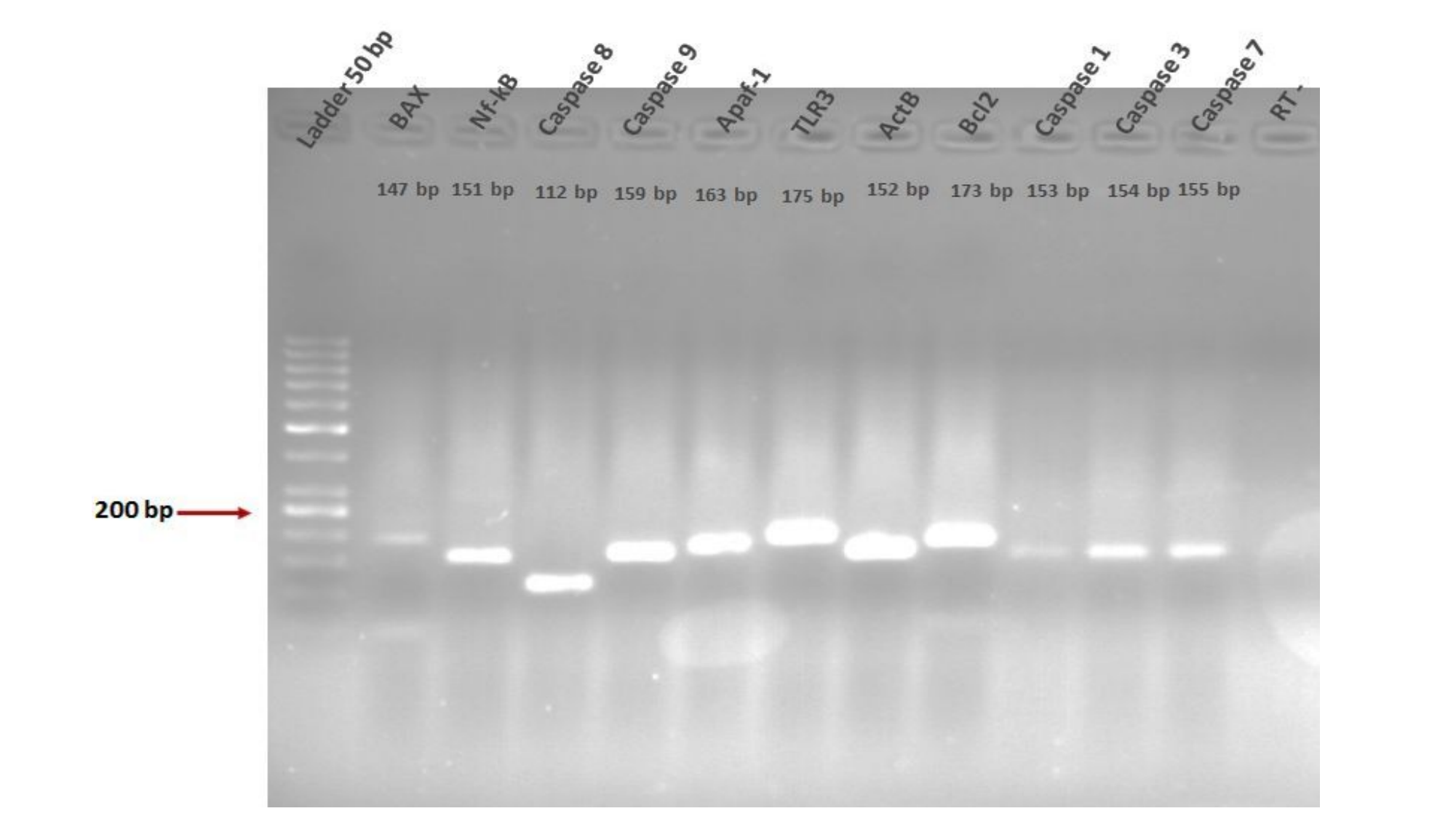

## Slide 2
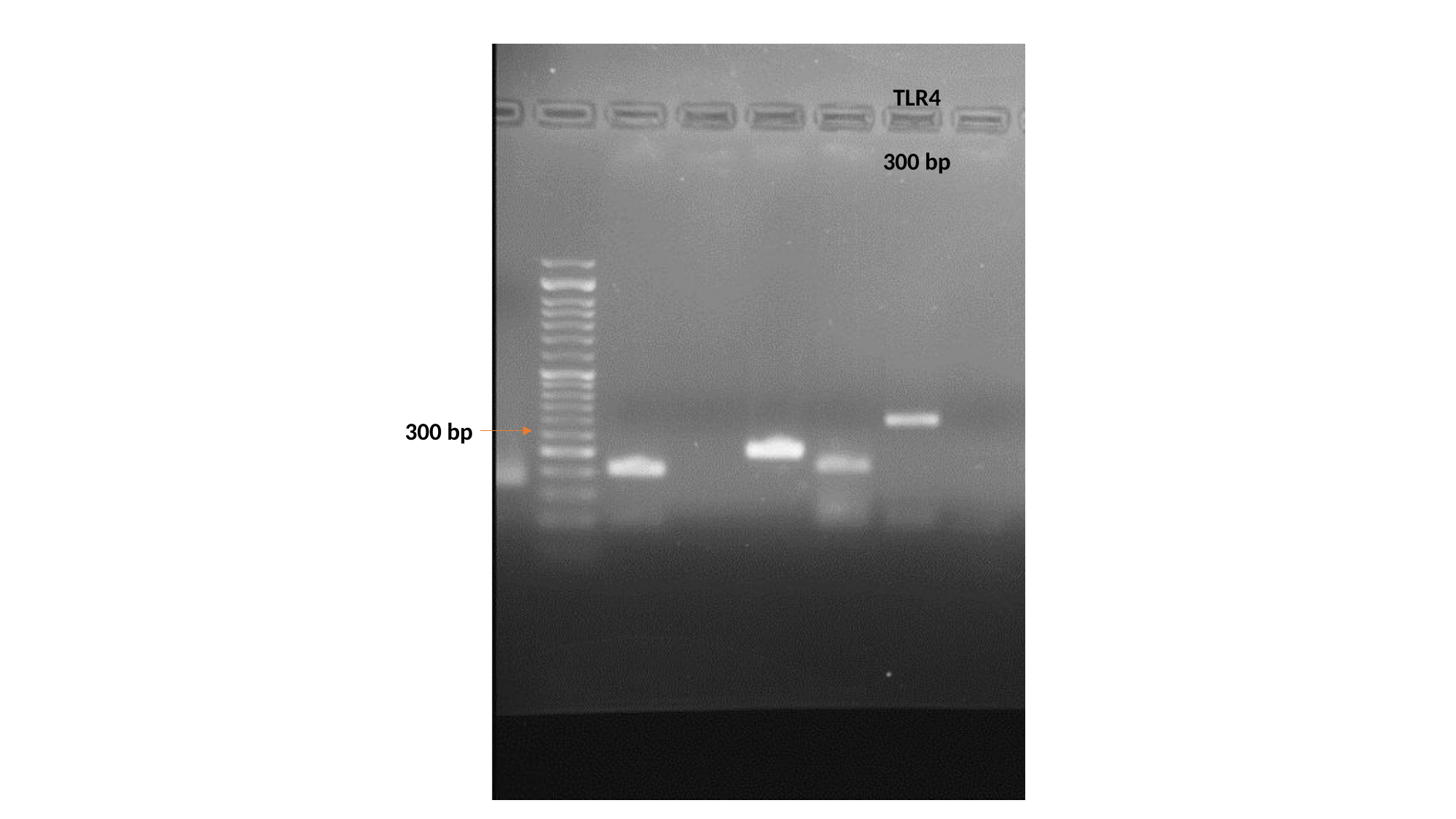

TLR4
300 bp
300 bp

## Slide 3
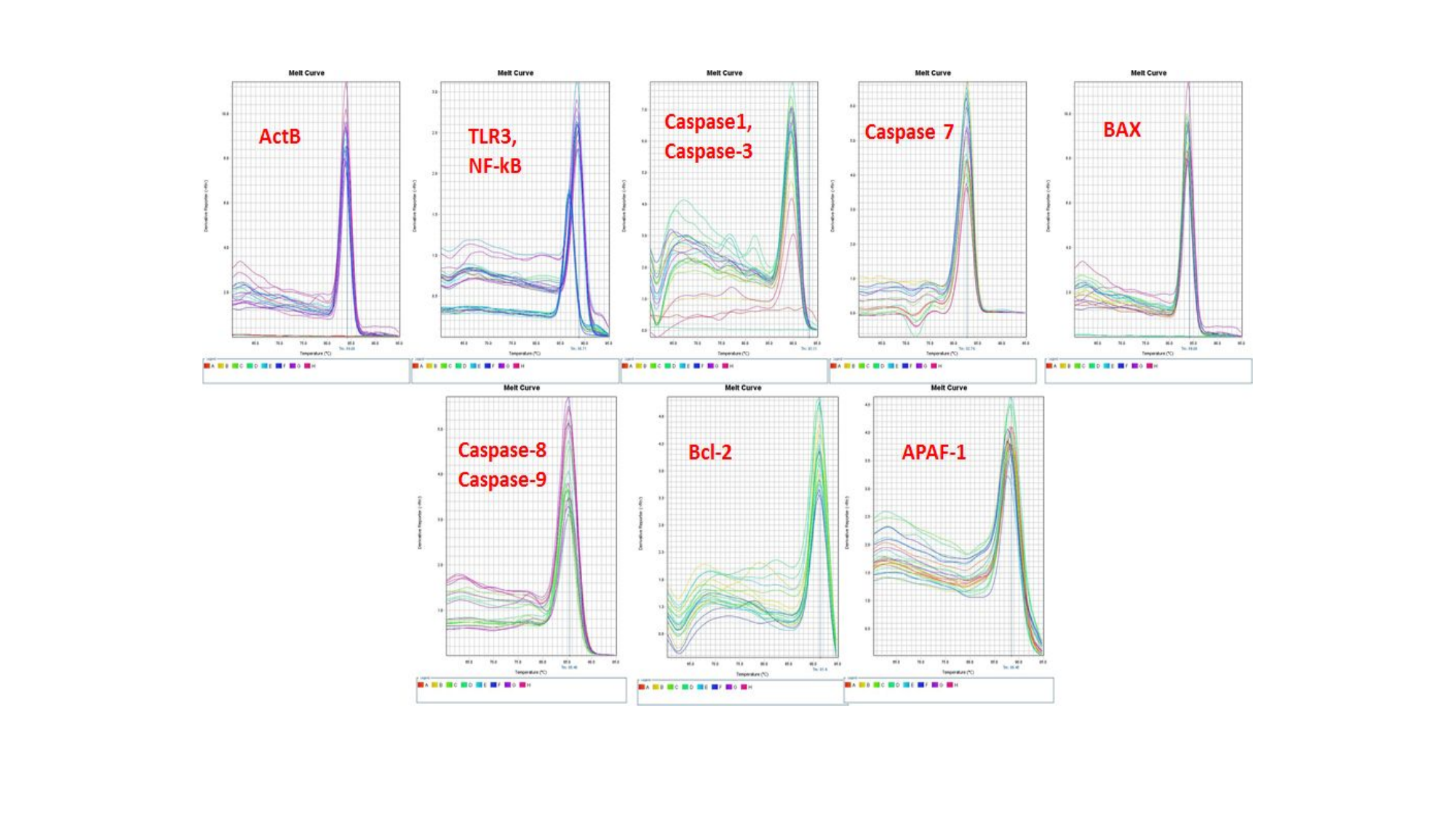

Supplement: Multimedia component 3 [file mmc3.pptx]
